# Pre- and post-procedural cardiac imaging (computed tomography and magnetic resonance imaging) in electrophysiology: a clinical consensus statement of the European Heart Rhythm Association and European Association of Cardiovascular Imaging of the European Society of Cardiology

**DOI:** 10.1093/europace/euae108

**Published:** 2024-05-14

**Authors:** Thomas Deneke, Valentina Kutyifa, Gerhard Hindricks, Philipp Sommer, Katja Zeppenfeld, Corrado Carbucicchio, Helmut Pürerfellner, Frank R Heinzel, Vassil B Traykov, Marta De Riva, Gianluca Pontone, Lukas Lehmkuhl, Kristina Haugaa, Andrea Sarkozy, Alessia Gimelli, Claudio Tondo, Sabine Ernst, Matthias Antz, Mark Westwood

**Affiliations:** Clinic for Rhythmology at Klinikum Nürnberg Campus Süd, University Hospital of the Paracelsus Medical University, Nuremberg, Germany; University of Rochester, Rochester, NY, USA; German Heart Center of the Charité, Berlin, Germany; Herz- und Diabeteszentrum NRW, Bad Oeynhausen, Germany; Department of Cardiology, Leiden University Medical Center (LUMC), Leiden, The Netherlands; EP and Arrhythmia Department, Centro Cardiologico Monzino IRCCS, Milan, Italy; Department of Clinical Electrophysiology, Ordensklinikum Linz Elisabethinen, Linz, Austria; Städtisches Klinikum Dresden, Department of Cardiology, Angiology and Intensive Care Medicine, Dresden, Germany; Department of Invasive Electrophysiology and Cardiac Pacing, Acibadem City Clinic Tokuda Hospital, Sofia, Bulgaria; Department of Cardiology, Leiden University Medical Center (LUMC), Leiden, The Netherlands; Department of Perioperative Cardiology and Cardiovascular Imaging, Centro Cardiologico Monzino IRCCS, Milan, Italy; Department of Biomedical, Surgical and Dental Sciences, University of Milan, Milan, Italy; Department of Radiology, Heart Center RHÖN-KLINIKUM Campus Bad Neustadt, Germany; Oslo University Hospital, Rikshospitalet, Oslo, Norway

**Keywords:** Cardiac computed tomography, Cardiac magnetic resonance imaging, Imaging-guided ablation, Imaging-aided ablation, Atrial fibrillation, Catheter ablation, Ventricular tachycardia, Active cardiac devices, Complications • Oesophago-atrial fistula

## Abstract

Imaging using cardiac computed tomography (CT) or magnetic resonance (MR) imaging has become an important option for anatomic and substrate delineation in complex atrial fibrillation (AF) and ventricular tachycardia (VT) ablation procedures. Computed tomography more common than MR has been used to detect procedure-associated complications such as oesophageal, cerebral, and vascular injury. This clinical consensus statement summarizes the current knowledge of CT and MR to facilitate electrophysiological procedures, the current value of real-time integration of imaging-derived anatomy, and substrate information during the procedure and the current role of CT and MR in diagnosing relevant procedure-related complications. Practical advice on potential advantages of one imaging modality over the other is discussed for patients with implanted cardiac rhythm devices as well as for planning, intraprocedural integration, and post-interventional management in AF and VT ablation patients. Establishing a team of electrophysiologists and cardiac imaging specialists working on specific details of imaging for complex ablation procedures is key. Cardiac magnetic resonance (CMR) can safely be performed in most patients with implanted active cardiac devices. Standard procedures for pre- and post-scanning management of the device and potential CMR-associated device malfunctions need to be in place. In VT patients, imaging—specifically MR—may help to determine scar location and mural distribution in patients with ischaemic and non-ischaemic cardiomyopathy beyond evaluating the underlying structural heart disease. Future directions in imaging may include the ability to register multiple imaging modalities and novel high-resolution modalities, but also refinements of imaging-guided ablation strategies are expected.

## Table of contents

1. Introduction
2. Basic concepts of computed tomography and magnetic resonance imaging
 2.1. Cardiac computed tomography
  2.1.1. Tissue imaging
  2.1.2. Post-procedural lesion imaging
 2.2. Cardiac magnetic resonance
  2.2.1. Tissue imaging
   2.2.1.1.Specific consideration of cardiac magnetic resonance sequences
    2.2.1.1.1. T1-weighted imaging
    2.2.1.1.2. T2-weighted imaging
    2.2.1.1.3. Extracellular volume
    2.2.1.1.4. First-pass gadolinium perfusion imaging
    2.2.1.1.5. Late gadolinium enhancement imaging
  2.2.2. Lesion imaging
 2.3. Workflow of image integration
3. Magnetic resonance imaging in active device patients
 3.1. Safety
 3.2.Image quality
4. Computed tomography and magnetic resonance imaging for atrial fibrillation ablation
 4.1. Pre-procedural imaging
 4.2. Association between imaging and AF ablation outcomes
 4.3. Intraprocedural image integration: role of CCT and CMR to aid/guide AF ablation procedures
 4.4. Left atrial and left atrial appendage fibrosis detection
 4.5. Optimal computed tomography imaging modalities for patients with atrial fibrillation
 4.6. Optimal cardiac magnetic resonance imaging modalities for patients with atrial fibrillation
 4.7. Important considerations for the use of cardiac computed tomography and cardiac magnetic resonance in patients with atrial fibrillation and procedures
5. Computed tomography/magnetic resonance for ventricular tachycardia procedures
 5.1. Role of cardiac computed tomography and magnetic resonance imaging in ventricular tachycardia ablation
  5.1.1. General recommendations
  5.1.2. Epicardial mapping and ablation
 5.2. Post-myocardial infarction cardiomyopathy ventricular tachycardia ablation
  5.2.1. Scar detection
  5.2.2. Real-time integration of imaging-derived scar
  5.2.3. Cardiac imaging of ventricular tachycardia channels
 5.3. Non-ischaemic cardiomyopathy ventricular tachycardia ablation
  5.3.1. Pre-procedural imaging/planning
  5.3.2. Real-time integration of imaging-derived scar
  5.3.3. Cardiac imaging for substrate-based ablation
 5.4. Arrhythmogenic right ventricular cardiomyopathy ventricular tachycardia ablation
  5.4.1. Pre-procedural planning
  5.4.2. Real-time integration of imaging-derived fibrofatty areas
 5.5. Optimal computed tomography imaging modalities for patients with ventricular tachycardia
 5.6. Optimal cardiac magnetic resonance imaging modalities for patients with ventricular tachycardia
 5.7. Important considerations for the use of cardiac computed tomography and cardiac magnetic resonance in ischaemic ventricular tachycardia ablation procedures
 5.8. Important considerations for the use of cardiac computed tomography and cardiac magnetic resonance in non-ischaemic ventricular tachycardia ablation procedures
6. Imaging for detection of ablation-related complications
 6.1. Oesophageal perforation after atrial fibrillation ablation
 6.2. Important considerations for the use of computed tomography and magnetic resonance in the diagnosis and management of oesophageal perforation
 6.3. Pulmonary vein stenosis after atrial fibrillation ablation
 6.4. Important considerations for the use of computed tomography and magnetic resonance in the diagnosis and management of pulmonary vein stenosis
 6.5. Neurological complications (stroke, Transient Ischemic Attack, and silent cerebral event/silent cerebral lesion) after ablation procedures
 6.6. Important considerations for the use of computed tomography and magnetic resonance in the diagnosis and management of neurological complications
 6.7. Complications related to vascular access
 6.8. Complications related to epicardial access
 6.9. Important considerations for the use of computed tomography and magnetic resonance in the diagnosis and management of vascular and epicardial access complications
7. Future aspects, studies, and concepts
Acknowledgements
Data availability

## 1. Introduction

Imaging has evolved as a cornerstone for the management of patients with complex arrhythmia substrates by helping to better understand the anatomy and the underlying structural abnormalities and to identify catheter treatment–related complications. In this regard, the current manuscript summarizes the current knowledge of cardiac computed tomography (CCT) and cardiac magnetic resonance imaging (CMR) to facilitate electrophysiological (EP) procedures. The current value of real-time integration of imaging-derived anatomy and substrate information during EP procedure and the role of CT and MR in diagnosing relevant procedure-related complications are reported.

The authors, as a joint group of electrophysiologists and experts in cardiovascular imaging, intend to provide practical advice on how to use CT and MR in different scenarios in patients with arrhythmia including patients with implanted active rhythm devices [cardiovascular implantable electronic device (CIED)]. It is intended to help electrophysiologists to decide on which technology and which specific techniques to use in clinical settings of atrial fibrillation (AF) and ventricular tachycardia (VT) ablation.

## 2. Basic concepts of computed tomography and magnetic resonance imaging

Echocardiography is the most used imaging modality for pre-procedural planning, intraprocedural monitoring, and post-procedural evaluation, because of absence of radiation, low costs, ready availability, and rapidity. However, CMR and CCT are valuable and may provide complimentary information. The choice of the imaging technique is determined by the indication, the specific advantages and limitations of the imaging modality, the availability, safety and convenience for the patient, and preferences and experiences of the physicians. In addition, when deciding between CCT or CMR, the existence of implanted cardiac devices in the target area (with potential for hindering artefacts) and patient baseline characteristics like renal and thyroid function need to be taken into account. Cardiac computed tomography but not CMR also implies radiation exposure. In regard to imaging the heart, an additional consideration is the higher spatial resolution in CCT vs. higher temporal resolution and most appropriate tissue characterization in CMR (see *Table 1*).

**Table 1:** Benefits and limitations of CCT and CMR

Considerations for using CMR vs. CCT
(1) Indication
(2) Availability/urgency (CCT > CMR)
(3) Need for higher spatial resolution (CCT > CMR)
(4) Need for higher temporal resolution (CMR > CCT)
(5) Need for most appropriate tissue characterization (CMR > CCT)
(6) Imaging limitations (especially artefacts from devices in CMR)
(7) Patient baseline characteristics (renal function, allergies, thyroid function)
(8) Radiation exposure (CCT)

Specific indications including benefits and limitations of the two imaging modalities useful to electrophysiologists for optimized implementation of imaging.

CCT, cardiac computed tomography; CMR, cardiac magnetic resonance imaging; > appears favourable/better.

In patients with congenital heart disease, imaging can create a three-dimensional (3D) roadmap to understand the complex anatomy. In these younger patients, free-breathing 3D CMR acquisition may be preferred over CCT as long contrast transit times may result in need for large amounts of contrast and long acquisition times exposing the patient to high radiation exposure.

Intraprocedural co-registration of electroanatomical mapping data with the 3D morphological reconstructions from CCT and/or CMR can facilitate mapping, may reduce the use of fluoroscopy during interventions, and may increase the safety for the patient and the operator.^1,2^

### 2.1. Cardiac computed tomography

Cardiac computed tomography is increasingly being implemented in clinical routine due to advances in the technology offering high spatial resolution and high diagnostic image quality. Recently introduced photon-counting detector CT is equipped with X-ray detectors which count the quantity and quality of incoming photons and photon energy allowing optimized spectral imaging capabilities with high temporal resolution. Further improvement in myocardial characterization in CCT may be achieved in high resolution, like iodine quantification in altered myocardium, the so-called iodine mapping.^3^

Pre-interventional CCT can be used to identify the (variation in) cardiac anatomy including pulmonary veins (PVs), non-invasive assessment of coronary artery anatomy and disease, and pulmonary pathologies. Computed tomography offers several advantages over echocardiography, as it provides high spatial resolution, is not related to specific echo windows of view, involves standardized measurements, and may use contrast-enhanced depiction of vasculature and chambers. It is the first-line imaging modality to non-invasively assess left atrial appendage (LAA) anatomy and size and can detect LAA thrombus.^4^ Cardiac computed tomography can identify PV stenosis and (asymptomatic) stenosis of coronary arteries. All cardiac and related structures can be segmented and made available during the ablation procedure (see image integration). Appropriate timing of contrast in the targeted chambers is important, and the window of imaging around the field may be modified to include relevant anatomic substrates (like the aortic arch in VT procedures) used for intraprocedural image registration (merging).

In VT procedures, pre-procedural CCT is an alternative to echocardiography and CMR for detecting underlying cardiac pathology and related abnormalities, such as ventricular thrombus.

In addition, CCT provides information on wall thickness, intramyocardial and epicardial fat, and myocardial calcification and, albeit with lesser accuracy than CMR, depicts delayed enhancement in myocardial scars. Wall thinning, e.g. after myocardial infarction (MI), can be visualized with a higher spatial resolution than CMR (CCT 128-slice scanner spatial resolution 0.4 mm; 1.5 T CMR spatial resolution 1.3 mm) and can be used for planning VT interventions.^5–7^ Left ventricular (LV) functional parameters can be determined reliably by CCT, but radiation exposure must be considered. Depending on the scanner technology used, regional wall motion abnormalities can be detected, but with lower temporal resolution compared to echocardiography or CMR.

In CCT, radiation exposure is of concern, especially when multiple image acquisitions are required. Radiation exposure has been reduced in the last decades and depends on body weight, heart rate and rhythm, tube voltage, and selected scan protocols^8–12^ (*Table 2*). Dose-saving protocols should be used whenever possible. Recent technological innovations have substantially reduced exposure to radiation. Acquisition modes with prospective electrocardiogram (ECG) triggering, including prospectively ECG-triggered high-pitch spiral acquisition, and the use of low kV protocols have led to a significant reduction in effective radiation doses.^8^ In principle, the use of modern scanner technology like 64-slice CT generation or higher is advised, similar to the advice for CT of the coronary arteries.

**Table 2:** Estimated and published effective patient doses (calculated using the chest coefficient (0.014) from dose length product)

Imaging technique	Approximated effective patient dose
CCT for LAA thrombus detection	1–4.7 mSv^a^
CCT angiography	1.5–4.7 mSv
CCT for tissue characterization	1.5–9 mSv
Coronary angiography	2–8 mSv
AF ablation	1.6–59.6 mSv
VT ablation	3.0–45.0 mSv

Approximated patient radiation exposure for different x-ray-based imaging modalities in cardiac imaging.^9–11^

AF, atrial fibrillation; CCT, cardiac computed tomography; LAA, left atrial appendage; mSv, millisievert; VT, ventricular tachycardia.

^a^highly dependent on scan protocol and triggering.

In general, effective patient doses appear to be lower in CCT for LAA thrombus detection compared to CCT angiography or CCT for tissue characterization but highly dependent upon triggering and scan protocols.^11^

#### 2.1.1. Tissue imaging

Cardiac computed tomography can be used for tissue characterization including late iodine enhancement, a methodology comparable to late contrast enhancement imaging using CMR.^13^ However, CCT has a limited contrast of myocardial scars and is inferior to scar depiction in CMR. A significant improvement in CT-based late enhancement may be achieved by using spectral CT and iodine maps (late iodine enhancement CCT). Scar delineation comparable to CMR has been described.^14^Cardiac computed tomography can detect fatty infiltrations, which have been correlated with low-voltage areas and VT-related sites in patients with arrhythmogenic cardiomyopathy (ACM).^15,16^ Cardiac computed tomography is sensitive for the detection of myocardial calcifications which may be relevant for VT ablation in patients with ischaemic cardiomyopathy (ICM).^17,18^

#### 2.1.2. Post-procedural lesion imaging

So far, no studies have evaluated post-procedural ablation lesion imaging using CCT.

### 2.2. Cardiac magnetic resonance

Cardiac magnetic resonance is a technique with excellent resolution and reproducibility, allowing for anatomical evaluations and functional studies. Specific acquisition techniques, including late gadolinium enhancement (LGE) imaging, allow for detailed tissue characterization. Cardiac magnetic resonance can be used to define the anatomy, to assess the (likely) underlying disease, and to detect procedure-related complications. In general, functional MRI sequences (cine) can be differentiated from static tissue characterizing sequences (e.g. black-blood sequences, mapping and/or LGE).

CMR studies are more time-consuming, are of relatively high costs regarding acquisition and personnel, and have lower scanner distribution compared to CCT. Cardiac magnetic resonance studies are limited by inadequate motion correction and require a relatively stable heart rhythm which can be an important limitation in patients with arrhythmia. Contrast studies with gadolinium agents may have a low but increased risk of toxicity in patients with severely impaired renal function, and indication should be carefully weighed.^19^ There are differences in image quality comparing 1.5 T MR scanners and 3 T scanners. In general, 1.5 T MR scanners are mainly used as standard for cardiac scanning, and rarely, 3 T scanners may be used for advanced imaging information.

The existence, potential compatibility, and resulting artefacts of active implantable cardiac devices (CIED) like pacemakers (PMs) or implantable cardioverter–defibrillators (ICDs) need to be considered when performing CMR. Estimations show that around half of PM and patients with ICD will require an MRI scan during the lifetime of their device, mainly for non-cardiac reasons.^20^ Most PMs are CMR conditional with 1.5 T MR scanners (information on 3 T scanners is scarce), but in particular, patients with ICDs or cardiac resynchronization therapy (CRT) devices may need specific management when undergoing CMR (see Section 3). Alternative image modalities should be considered in patients with non-compatible devices.

#### 2.2.1. Tissue imaging

In general, CMR is considered the gold standard for tissue characterization and enables visualization of ablation lesions. In the acute stage of myocardial damage, CMR identifies oedema, necrosis, and intramyocardial haemorrhage, whereas in the chronic state (after months), it identifies myocardial scar and fibrosis. Quantitative parametric mapping imaging may lead to less inter-operator variability compared to pure qualitative analysis. There are different scanning sequences that may be helpful to differentiate between different degrees and acuity of myocardial damage (see *Table 3*).

**Table 3:** Relevance of different scanning sequences of CMR in regard to detection of myocardial pathology

		Descriptive results			Disease/entity		
	Normal	Oedema	Regional fibrosis/scar	Diffuse fibrosis	Myocarditis	Acute ablation lesion	Chronic ablation lesion
T1	0	+	+	+	+	+ (TWILITE)	+
T2w	0	+	0	0	+	+	0
ECV	0	+	+	+	+	+	+
LGE	0	0	+	0	0	0	+

ECV, extracellular volume; LGE, late gadolinium enhancement; TWILITE, T1-weighted long inversion time sequence; T2w, T2-weighted.

Whereas LGE sequences provide information on irreversible myocardial damage and scar, novel techniques with T1 and T2 mapping and assessment of extracellular volumes (ECVs) have an established role for tissue characterization by CMR and can be useful for diagnostic considerations.^21^ These techniques can identify oedema and increased interstitial volume, which is not necessarily due to fibrosis, and are independent of whether myocardial disease is focal or diffuse. T1 relaxation times decrease by fat or iron infiltration and increase by fibrosis and amyloid, while T2 relaxation times increase by oedema. Based on T1 mapping in patients receiving gadolinium agents, ECV can be assessed and provides a good estimate of diffuse fibrosis. There are currently technical limitations including CMR system-related variability and issues of normal/reference ranges. The clinical value of these techniques in the setting of EP interventions requires further studies.^22,23^

Continuously adaptive windowing strategy has been described as a fully automated, fast, and efficient technique for high-resolution free-breathing acquisition. It allows acquisition of the entire blood pool free breathing and shortens scan times while generating high-resolution non-contrast 3D image quality.^24^

##### 2.2.1.1. Specific consideration of cardiac magnetic resonance sequences

###### 2.2.1.1.1. T1-weighted imaging

The longitudinal relaxation T1 is defined as time required for longitudinal magnetization to recover from the transverse plane to 63% of its value after 90° excitation. Any increase in interstitial space e.g. from oedema or diffuse fibrosis causes elevated T1 relaxation times. Water has a slow longitudinal magnetization resulting in long T1 relaxation times and thereby appears dark on CMR. Denaturized proteins within necrotic areas and conversion of ferrous iron in myoglobin and haemoglobin may account for shorter T1 relaxation times. Native, non-contrast agent-enhanced T1-weighted (T1w) imaging can visualize acute myocardial damage related to ablation (within the first hours). T1 relaxation times are longer than normal in hypertrophic myocardium (hypertrophic cardiomyopathy), inflammation/myocarditis, and amyloidosis (global increase), whereas they are shorter than normal in fatty dysplasia, iron overload, and Morbus Fabry.^25^ T1-weighted long inversion time imaging allows imaging of acute ablation lesions without using contrast agents.

###### 2.2.1.1.2. T2-weighted imaging

The transverse relaxation T2 is defined as the time required for transverse magnetization to decay to 37% of its value after 90° excitation. Tissue with high water content has prolonged T2 relaxation times and therefore shows up as increased signal intensity (SI) on T2-weighted (T2w) images. Therefore, T2w imaging allows identification of oedema, visible as bright high-intensity signal, or inflammation (myocarditis).

T2-weighted imaging techniques have a low spatial resolution, high sensitivity to cardiac motion, and arrhythmias, which may lead to non-uniform signal detection and may reduce reproducibility and reliability.

###### 2.2.1.1.3. Extracellular volume

Expansion of the ECV may occur secondary to fibrosis. The calculation of ECV is based on the observation that gadolinium contrast in the interstitium shortens T1. The ratio of pre- and post-contrast T1 of the myocardium and blood corrected for haematocrit is evaluated. Extracellular volume can be longitudinally followed and compared and is usually used for tracking diffuse myocardial fibrosis.

###### 2.2.1.1.4. First-pass gadolinium perfusion imaging

First-pass perfusion imaging is used to evaluate myocardial blood flow and perfusion and involves injection of a contrast medium and early imaging during the first pass of the contrast medium. First-pass perfusion is used to identify distribution and differences in blood flow within the myocardium at rest and during hyperaemia. Perfusion defects indicating coronary artery and also microvascular obstruction can be identified and may be seen in the early stage of myocardial damage.

###### 2.2.1.1.5. Late gadolinium enhancement imaging

Late gadolinium enhancement (LGE) imaging is based on the delay of contrast agent wash out of the myocardium with a high proportion of extracellular space. This is typically found in focal fibrosis, inflammation, or fat infiltration but also as a result of ablation-induced chronic fibrosis and inflammation. Late gadolinium enhancement imaging identifies irreversible myocardial damage.

Late gadolinium enhancement is visualized by T1w imaging 10–20 min after contrast injection. Sequential two-dimensional (2D) images are usually used to identify LGE in the ventricle, but 3D high-resolution sequences are available. Higher concentrations of gadolinium appear bright in LGE-CMR. In acute myocardial damage, dark zones may be identified within LGE areas characterizing relevant microvascular injury that leads to intramural haemorrhage and aggregation of erythrocytes outside the vasculature.

Late gadolinium enhancement can also be assessed in the atria although this image modality is hampered by the spatial resolution for the thin atrial wall. Atrial LGE may be of prognostic value for the outcome of AF ablation success,^26^ but atrial imaging using MR, especially LGE, remains controversial and reproducibility remains an issue.

#### 2.2.2. Lesion imaging

Imaging to document ablation lesions has mostly been performed in patients after AF ablation using CMR technology. Radiofrequency (RF) ablation results in a variety of changes on LGE with hyper- or non-enhancement. In patients undergoing immediate post-ablation LGE, non-enhancement lesions demonstrate no-reflow characteristics and may allow prediction of definitive scar formation after AF ablation.^27^ Current data suggest that 3-month LGE imaging best characterizes chronic RF ablation-induced atrial scar formation.^27,28^

In ventricular myocardium, CMR may also detect RF ablation lesions within 3 days after the procedure as non-enhanced lesions during early gadolinium enhancement scans (3 min after gadolinium contrast injection) surrounded by hyperenhanced zones resembling the no-reflow phenomenon observed in patients with acute stages of MI. The size and depth of early non-enhanced lesions appear to correlate with the ablation energy and impedance drop during ablation and may not be associated with acute success.^29^ Late gadolinium enhancement months after an ablation procedure have identified hyperenhanced lesions in approximately two-thirds of patients.^30^ The translation of these findings into efficacy of EP procedures requires further studies and may also involve data from real-time MRI-guided ablation.^31^

### 2.3. Workflow of image integration

Cardiac computed tomography or CMR data sets can be imported into electroanatomic mapping systems (EAMS). There are specific preconditions for image acquisition depending on the EAMS (Digital Imagin and Communications in Medicine; DICOM format). The structures of interest can be identified and separated from each other and from the remaining imaged structures (process of SEGMENTATION). In the EAMS, segmentation can be performed semiautomatically or automatically via seeding. Different algorithms and work processes exist in the different EAMS. Manual editing is possible and may be needed. Segmentation of cardiac and related structures can also be performed using image processing software and uploaded into the EAMS either in DICOM or presegmented VTK files. The 3D reconstruction of the segmented structures of interest can be displayed in the electroanatomic mapping (EAM) system. Integration of segmented images with EAM data can be achieved either by the alignments of landmarks which can be tagged during catheter mapping and are also identifiable on imaging or by surface registration of the 3D anatomical (endocardial) shell of the mapped chamber(s) of interest and the segmented imaging-derived contours (REGISTRATION). In most cases, a mix of both techniques is applied and may warrant higher precision of registration.

Some portions of the mapped structures may be susceptible to deformation when approached with catheters which may affect anatomy. Respiratory phase filter, gating per beat, and exclusion of premature beats (e.g. premature ventricular complexes) are applicable to all EAM systems and may help to generate more reliable anatomical maps. Manual correction may be needed. Differences of registration processes exist between current 3D mapping systems, CARTO (CARTOMERGE), EnSite (NavX Fusion), and Rhythmia (Rhythmia automated alignment) and may affect registration accuracy. So far, no comparative study exists. The registration process is critical if imaging-aided or imaging-guided approaches are intended. The more accurate the registration process, the more operators can rely on the integrated imaging information.

## 3. Magnetic resonance imaging in active device patients

Cardiac magnetic resonance can safely be performed in the majority of patients with CIED.^32–36^ While there is growing evidence on the safety of MR in patients with MR-conditional and non-conditional devices as well as in patients with abandoned leads, no data on patients with fractured leads or lead extenders are available. Cardiovascular implantable electronic devices introduce susceptibility artefacts that may preclude analysis of MR images. Close coordination between imaging and CIED experts to ensure proper pre-, intra-, and post-scanning management of patients with CIED is important. Careful selection of the needed imaging modality (CMR vs. CCT) and shared decision making is key. All medical information on CIEDs including leads, potentially abandoned or fractured leads, and lead extenders should be collected. Magnetic resonance-conditional CIEDs are only tested for 1.5–3 T MR scanners, and 1.5 T CMR may be selected for non-conditional devices. Cardiac computed tomography may also display artefacts from CIED scans and electrodes.^32^ Manufacturers of CIEDs have developed newer devices with non-ferromagnetic components and have improved shielding to allow MR imaging.^33–38^

### 3.1. Safety

**Figure 1:**
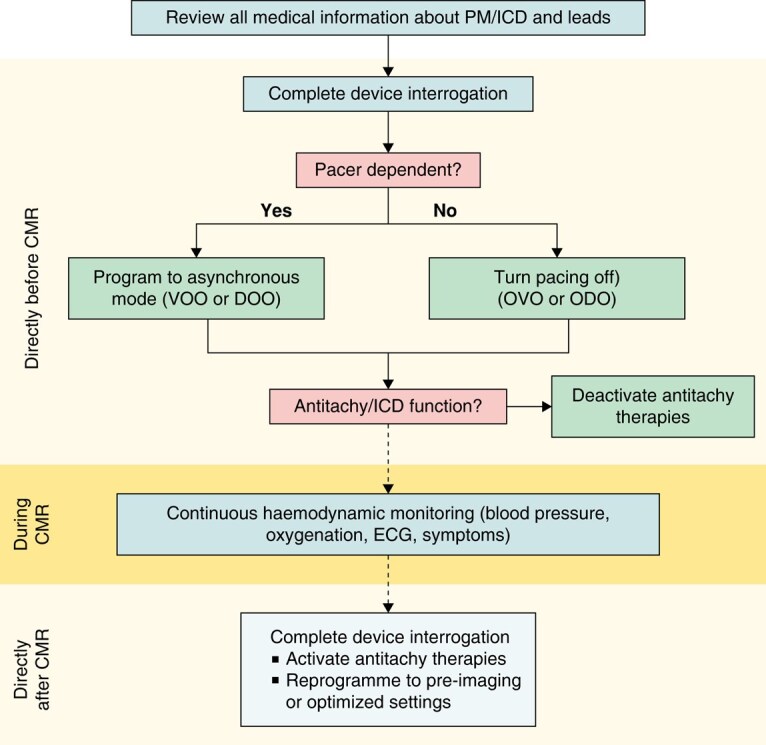
Standardized CMR protocol for patients with PM or ICD undergoing a 1.5 T CMR at timepoint before, during, and after CMR. CMR, cardiac magnetic resonance imaging; ECG, electrocardiogram; ICD, implantable cardioverter–defibrillator (modified from^43^); PM, pacemaker.

Data from multiple registries support the safety of CMR imaging in patients with CIED with extremely low complication rates.^32–36,39–42^ However, adherence to standard operating protocols is strongly advised.^38,43^ Important aspects are patient selection, patient information and consensus, device interrogation and programming before CMR, subsequent device interrogation, and re-programming after CMR (*Figure 1*). There is an ongoing debate regarding CMR in patients with abandoned leads. While abandoned leads potentially lead to tissue heating at the tip of the lead, registry data indicate that CMR imaging may also be safe in these patients with low complication rates.^44,45^ Newer CIEDs are often labelled as ‘MR-conditional’, while older devices are not. This implies that such devices have a so-called MR mode which can be used to programme settings that are deemed preferable during MR scanning by the device manufacturer. Real-world data show that even in devices that do not have this option, it is possible to programme pacing modes that are well suited for CMR scanning. A combination of components from different manufacturers of CIEDs has not been tested and therefore cannot be declared MRI-conditional (*Table 4*).

**Table 4:** Specifications, definitions, and management strategies for patients with CIED undergoing MR procedures

Device specifications	Definitions	Advice for MRI
**MRI-conditional**	All components of the device are tested as MRI-conditional	Cleared for 1.5 T and if specified also for 3.0 T MRI, monitored setting advised
**MRI-non-conditional**	Manufacturer not cleared for use in MRI conditions, combination of parts of different manufacturers	1.5 T MRI may be performed, precautions and monitoring advised
**MRI-unsafe**	Fractured leads, lead extenders	No data on MRI safety available

MRI, magnetic resonance imaging.

Time in the scanner should be kept as short as possible to reduce the potential for interferences. It is advised to perform device interrogation and programming immediately before and after CMR. An expert in device handling and trained in advanced cardiac life support should be stand-by for emergency treatment during the scan. In the case of generator malfunction, standard operating procedures on evacuation of the patient from the scanner and how to perform emergency care like defibrillation or assure ventricular capture pacing need to be established.

Patients with need for ventricular stimulation and those with need for ICD interventions are specifically in need of close follow-up strategies, and remote monitoring may become essential. Patients with CIEDs should be rhythm-monitored throughout the time period within the scanner and until reprogramming of the device. In Implantable Loop Recorders (ILR) patients, stored data should be interrogated before scanning as otherwise this information could get lost.

### 3.2. Image quality

**Figure 2:**
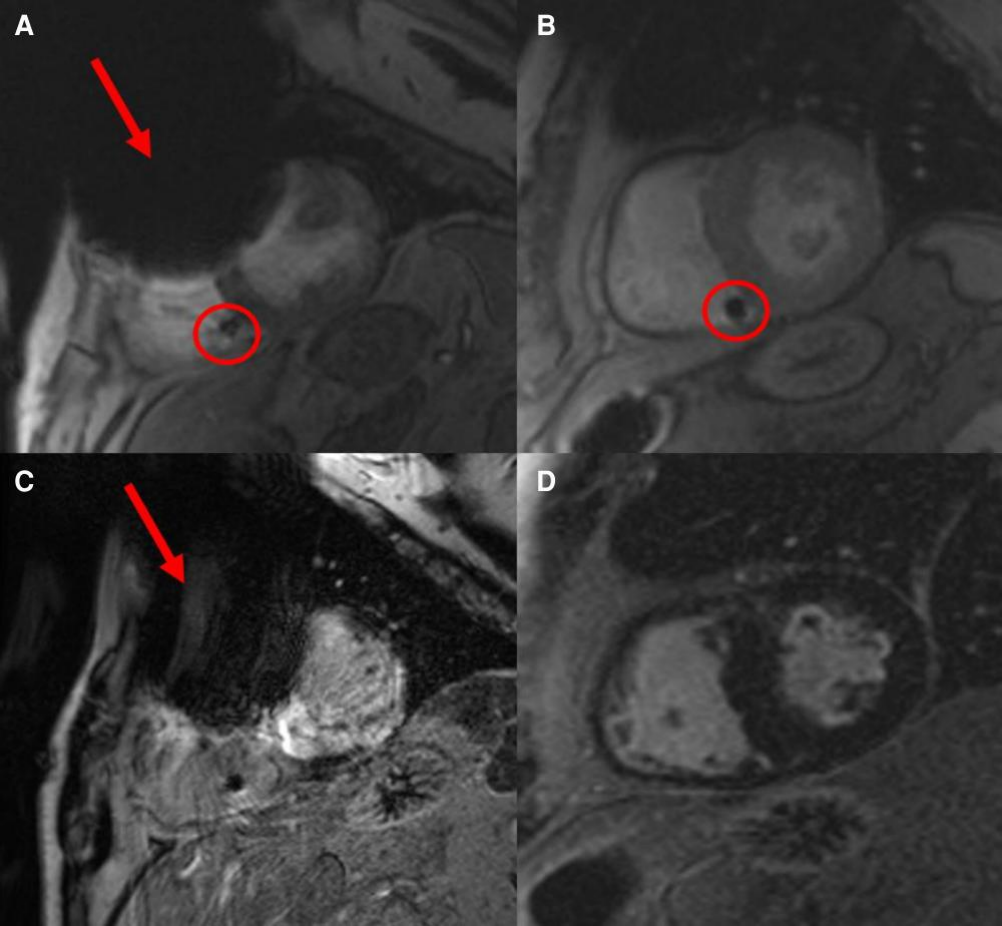
Wideband sequences (*B* and *D*) suppressing device-related artefacts (arrows): two patients with implanted ICDs [*A*, *C* Patient 1 imaged without wideband sequences showing relevant device-related artefacts (red arrows) and *B*, *D* Patient 2 imaged with wideband sequences]. *A*, *B* Cine sequence and *B*, *D* LGE imaging. circle: RV device lead. ICDs, implantable cardioverter–defibrillators; LGE, late gadolinium enhancement; RV, right ventricular.

Cardiovascular implantable electronic devices are made of various metals and impair CMR images by introducing artefacts. Depending on the device manufacturer, type of CIED (ILR vs. PM vs. ICD), device position, and number of connected leads, artefacts greatly vary in size and location. Both the generator and the leads introduce loss of signal and hyper-intensity artefacts. While signal loss is easily identifiable, hyper-intensity artefacts need to be carefully evaluated to avoid false interpretation (e.g. as fibrosis) in LGE images. In general, a generator that has been implanted on the right side is less likely to produce relevant artefacts compared to left-sided generators. Patients with left pectoral CIEDs are most prone to artefacts. The distance between the lower edge of the generator and the heart’s silhouette may determine artefact intensity. Changing patient location within the scanner may help to manipulate the device can out of the area of interest. The most extensive artefacts and least number of evaluable cardiac segments on CMR are seen in CRT–ICD devices. Artefacts usually are focused on the anterior and septal portion of the heart. Different sequences may have different severity of artefacts being lower in black-blood sequences than in LGE sequences and highest in cine images.^26^ Fast gradient echo sequences for cine and wideband sequences for LGE (*Figure 2*) may reduce artefacts.^33,37^

## 4. Computed tomography and magnetic resonance imaging for atrial fibrillation ablation

Advanced imaging modalities provide information about PV and left atrial (LA) anatomy, help to detect LAA thrombus, and may aid in individual risk stratification for thromboembolism and AF recurrences after ablation.

### 4.1. Pre-procedural imaging

Cardiac computed tomography and CMR systematically detect higher LA volume compared with 2D echocardiography with a trend of overestimation with CCT as compared to CMR. No differences are described in terms of diagnostic accuracy of PV patterns between the two imaging modalities.^46,47^

Cardiac computed tomography and CMR are accurate in delineating LA anatomy and are able to categorize LAA morphologies as cactus, chicken wing, windsock, or cauliflower pattern^48^ with relevance to risk of stroke (cauliflower morphology was associated with an 8.0-times higher likelihood of stroke compared to chicken wing morphology).^48^ Characterization of LAA morphology may therefore be additional helpful information for stratifying stroke risk.

**Figure 3:**
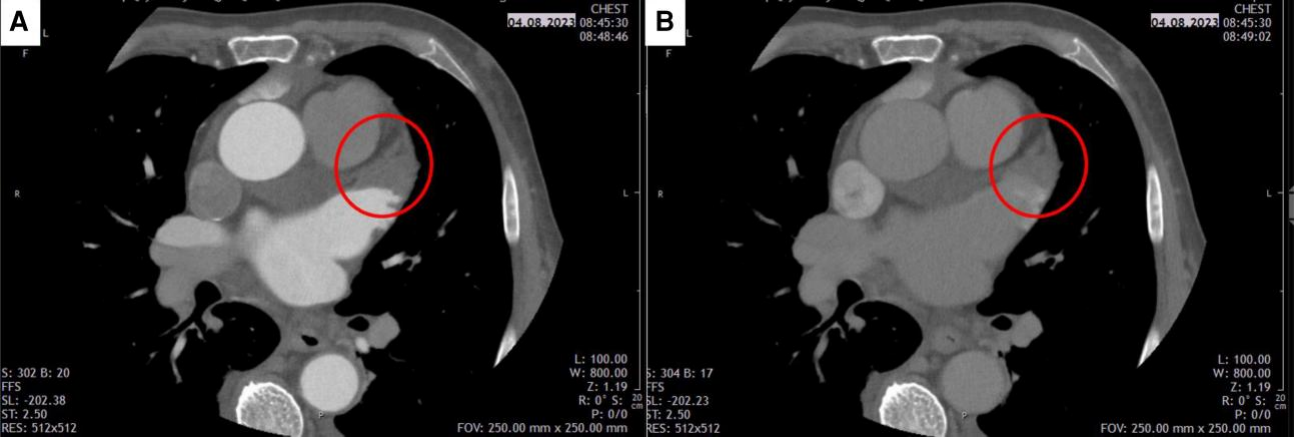
Left atrial appendage thrombus (circle) in early arterial (*A*) and early venous (later) phase (*B*) CCT imaging. CCT, cardiac computed tomography.

Cardiac computed tomography and CMR are able to rule out LAA thrombus with a high sensitivity and specificity. The diagnostic accuracy of CCT vs. transoesophageal echocardiography (TOE) is 94%. Delayed imaging on top of arterial phase acquisition in CCT increases the positive predictive value to 92% with an overall diagnostic accuracy of 99% (see *Figure 3*).

Cardiac magnetic resonance is equally effective in assessing LAA thrombus as compared to TOE with inversion time myocardial delayed enhancement (MDE) acquisition having the highest diagnostic accuracy (99.2%), followed by contrast-enhanced CMR angiography (94.3%) and cine CMR (91.6%).^49^

The oesophagus can be imaged using CCT and CMR. The right pericardiophrenic artery can be visualized by CCT to locate the right phrenic nerve potentially helping to identify patients at risk of phrenic nerve injury during Pulmonary Vein Isolation (PVI).^50,51^ Cardiac computed tomography and CMR can visualize the oesophagus, but the variability of the oesophagus position due to its mobile nature limits their intraprocedural use.^52^

### 4.2. Association between imaging and AF ablation outcomes

Several studies have shown an association between the amount of epicardial fat and outcomes after AF ablation. The most accurate technique for quantification is by volumetric quantification^53^ by CCT or CMR. Whereas CCT can detect epicardial fat with high reproducibility (contrast attenuation ranges between −195 and −45 HU), it may be appropriate to consider CMR as the true ‘gold standard’ as it is the only imaging modality that has been validated ex vivo.^54^

In CMR, LV myocardial native T1 time was greater in patients with AF conferring a six-fold increased risk of AF recurrence.^55^ Patients with LV-LGE had a two-fold higher rate of AF recurrence compared to patients without.^56^ In regard to recurrent AF, there are a positive association with LV-ECV, LA volume, and LV mass and a negative association with diastolic function.^57^

### 4.3. Intraprocedural image integration: role of CCT and CMR to aid/guide AF ablation procedures

Two single-centre observational studies^58,59^ suggested a superior efficacy of pre-acquired imaging integration for catheter ablation of AF. In both studies, a shorter procedure duration and superior outcome were documented when image integration was used. Two randomized trials^60,61^ showed no benefit in regard to rhythm outcome. The CAVERN trial^59^ compared image integration using either the Carto or the NavX system and found no difference in terms of freedom from arrhythmia but faster 3D image registration, lower fluoroscopic dose, and overall procedural time with Carto system as compared to NavX system. A recent meta-analysis of comparative trials did not identify an effect of image integration on AF ablation outcome.^62^

### 4.4. Left atrial and left atrial appendage fibrosis detection

Atrial fibrosis is the consequence of several individual and multifactorial processes. It is involved in the occurrence and perpetuation of focal and re-entry arrhythmia mechanisms^63,64^ as well as a major contributing factor to AF occurrence and persistence. Cardiac magnetic resonance has been used to detect location and degree of LA fibrosis, but no data on CCT to image atrial fibrosis are available. Post-ablation CMR may allow to also detect residual fibrosis and non-effective PVs isolation, both strong predictors of arrhythmia recurrence.^28,65–67^

In the DECAAF I study,^68^ LA fibrosis was evaluated with high-resolution 3D LGE respiration-navigated and fat-saturated sequence in order to develop the Utah score based on the amount of LA wall enhancement expressed as a percentage of the total LA wall surface: stage I, defined as <10%, stage II ≥10–<20%, stage III ≥20–<30%, and stage IV ≥30%. Left atrial tissue fibrosis was associated with the likelihood of recurrent atrial arrhythmias.

King *et al.*^69^ found a 1.67 hazard ratio comparing patients with stage IV vs. stage I for the composite arrhythmic events. However, LA wall image intensity on LGE is influenced by several parameters such as amount of gadolinium contrast, surface coil proximity, delayed time of image acquisition, patient haematocrit, glomerular filtration rate, and body mass index.^70^ In order to standardize LA fibrosis quantification, LGE analysis technique has been normalized by blood pool intensity.^70^

The LAA has been reported to be an under-recognized trigger site for AF. Patients with a high LGE extent involving the LAA have an approximately four-fold increased risk of AF recurrences compared to patients without LAA involvement.^59^ A limitation is the reproducibility of LA fibrosis detection especially for small scar areas.^71^

**Figure 4:**
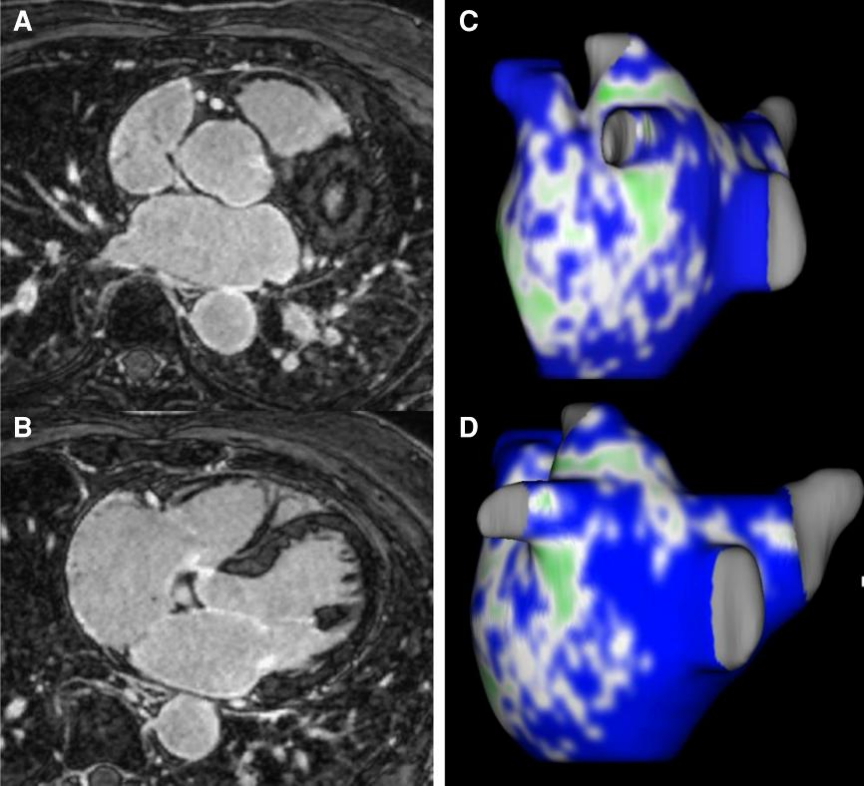
Example of 3D LA reconstruction with contrast-enhanced CMR angiography (*A*, *B*) and 3D LA fibrosis reconstruction (*C*, *D*) (using Merisight technology) (green: dense fibrosis; blue: normal atrial myocardium). CMR, cardiac magnetic resonance; LA, left atrial.

Processed data of LA fibrosis can be integrated into EAM to guide AF ablation. This concept was tested in the controlled randomized DECAAF II study but did not show benefit related to rhythm outcome in cases with persistent AF (see *Figure 4*).^72^

Late gadolinium enhancement imaging 3 months after ablation has been used to identify gaps in ablation lines and guide redo procedures to terminate LA macro-re-entrant tachycardias.^28,73^ It appears that CMR imaging 3 months after AF ablation best describes chronic ablation scar formation and may be helpful for redo procedure planning and guidance.

Cardiac magnetic resonance scans after pulsed field ablation for AF have shown different patterns of LGE and T2w images with large acute LGE volume and less oedema (in T2w imaging) without microvascular damage or intramural haemorrhage, whereas at 3 months most LGE had disappeared.^74^

### 4.5. Optimal computed tomography imaging modalities for patients with atrial fibrillation

Computed tomography imaging of the left atrium may differ from the standard CCT approach used to image coronary anatomy. On the one hand, the contrast medium dynamics in the left atrium and LAA are different, and on the other hand, the left atrium is generally exposed to movement artefacts to a much lesser extent due to cardiac pulsation than the coronary arteries. It is advised to acquire an early arterial phase, in which the LAA is normally not yet fully contrasted and subsequently an early venous phase including only the LAA later after contrast injection (usually <60 s). In this phase, the LAA should be completely contrasted if no thrombus is present. Due to the only modest movement of the left atrium during the cardiac cycle, it is in principle possible to completely dispense ECG triggering for LA imaging if fast scanning protocols are used (e.g. high-pitch mode). This enables a reduction in the amount of contrast medium required and shortens the examination time.

Axial thin-slice image reconstructions are used for 3D planning data sets. Three-dimensional reconstructions of the left atrium are helpful for the depiction of accessory PVs (*Table 6*).

### 4.6. Optimal cardiac magnetic resonance imaging modalities for patients with atrial fibrillation

The CMR minimum requirement is a 1.5 T scanner with a phased-array coil system. Several protocols have been described to evaluate LA and right atrial chamber anatomy, PV anatomy, and LAA morphology using late gadolinium-enhanced MR angiography.^75^ In addition to LGE acquisition, MDE obtained approximately 10 min after intravenous gadolinium contrast administration using a long inversion time or a 3D MDE fat saturation sequence with navigator is required to rule out LAA thrombus and LA fibrosis, respectively^49^ (see *Figure 3*). Late gadolinium enhancement can be assessed in the atria although this image modality is hampered by the spatial resolution for the thin atrial wall. Atrial LGE may be of prognostic value for the outcome of AF ablation success^26,76^ (*Table 5*).

**Table 5:** Advice table for CMR imaging in active device patients

Advice for MR imaging in active device patients	Strength of advice
(1) It is advised to carefully evaluate indication to perform CMR in patients with CIED	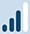
(2) In patients with CIEDs CCT may be preferred to CMR if primary anatomic information is warranted	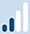
(3) CMR may be appropriate in patients with CIED to specify the underlying cardiac abnormalities and target regions for VT ablation	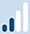
(4) A dedicated team of imaging specialist and electrophysiologist may be implemented to consensus on indication, optimum mode of imaging, goal of imaging, and need for follow-up	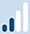
(5) CMR in patients with CIED is advised to only be performed if an expert in device handling is available on site	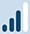
(6) Complete interrogation of PMs and ICDs is advised to be performed directly before and after CMR imaging	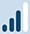
(7) Emergency standard operating procedures for device malfunction during CMR are advised to be in place	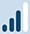
(8) Close follow-up of patients with CIED after CMR following a predefined plan is advised to identify potential malfunction, most effectively using frequent remote monitoring	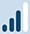

CIED, cardiovascular implantable electronic device; CCT, cardiac computed tomography; CMR, cardiac magnetic resonance imaging; ICD, implantable cardioverter–defibrillator; MR, magnetic resonance; PM, pacemaker; VT, ventricular tachycardia.

**Table 6:** Advice table for the use of CCT and CMR in patients with AF and procedures

Advice for the use of CCT and CMR in patients with AF and procedures	Strength of advice	Imaging modality	CT specifications	MR specifications
(1) If imaging is available, LAA morphology is advised to be categorized to assess stroke risk	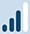	CCT or CMR	i.v. contrast injection and imaging in arterial phase	CEMRA
(2) CCT or CMR may be appropriate to assess LA and PV anatomy which may be integrated into the mapping system	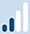	CCT or CMR	i.v. contrast injection and imaging in arterial phase	CEMRA
(3) Imaging may be an appropriate alternative to TOE to rule out LAA thrombus	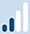	CCT or CMR	i.v. contrast injection and imaging in arterial and delayed phase	Inversion time MDE, CEMRA and cine CMR
(4) Degree of atrial fibrosis may be measured by CMR to identify the appropriate candidate for ablation and to guide treatment options	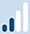	CMR	—	3D inversion time MDE navigator fat sat

AF, atrial fibrillation; CT, computed tomography; CCT, cardiac computed tomography; CEMRA, contrast enhancement magnetic resonance angiography; CMR, cardiac magnetic resonance imaging; LAA, left atrial appendage; LA, left atrium; MDE, myocardial delayed enhancement; MR, magnetic resonance; PV, pulmonary vein; TOE, transoesophageal echocardiography.

### 4.7. Important considerations for the use of cardiac computed tomography and cardiac magnetic resonance in patients with atrial fibrillation and procedures

1. Cardiac computed tomography and CMR are accurate in delineating LA anatomy and are able to categorize LAA morphologies as cactus, chicken wing, windsock, or cauliflower pattern.^48^ Cauliflower morphology was 8.0 times more likely to have had a stroke vs. chicken wing morphology.^48^ Characterization of LAA morphology can be helpful information and may be integrated into stroke risk assessment, but consequences regarding oral anticoagulation need to be determined.
2. Cardiac computed tomography has a higher spatial resolution than CMR in delineating LA and right atrial anatomy. No differences are described in terms of diagnostic accuracy of PV patterns between the two imaging modalities.^46,47^ A recent meta-analysis of comparative trials did not identify an effect of image integration on AF ablation outcome.
3. Cardiac computed tomography and CMR are useful to rule out LAA thrombus. Cardiac computed tomography has a documented diagnostic accuracy of 94% vs. TOE. Delayed imaging (venous phase) in addition to early arterial phase acquisition in CCT increases the positive predictive value to 92% with an overall diagnostic accuracy of 99%. Cardiac magnetic resonance is equally effective in assessing LAA thrombus compared to TOE with inversion time MDE acquisition having the highest diagnostic accuracy (99.2%), followed by contrast-enhanced CMR angiography (94.3%) and cine CMR (91.6%).^49^
4. Cardiac magnetic resonance has been used to detect location and degree of LA fibrosis, but no data on CCT to image atrial fibrosis are available. Late gadolinium enhancement-quantified relative LA fibrosis was used in the Utah score and affects AF ablation efficacy. Late gadolinium enhancement-guided LA fibrosis ablation was not superior to PVI in patients with persistent AF.^15,77,78^

## 5. Computed tomography/magnetic resonance for ventricular tachycardia procedures

There is unambiguous evidence that most re-entry VT-related sites in different structural cardiac abnormalities arise from scar as detected by imaging. Late gadolinium enhancement appears to be the superior and most studied modality to identify myocardial scar. Post-processing approaches either categorize tissue into scar vs. normal myocardium or aim to identify scar core and border zone based on SI thresholds. Different methods and SI thresholds have been applied, which may affect scar delineation. Cardiac computed tomography has also been used, particularly in patients with contraindications for CMR, to identify wall thinning, delayed enhancement, or fat infiltration. Cardiac computed tomography has a significantly higher spatial resolution, but drawbacks are the unfavourable signal-to-noise ratio with suboptimal results particularly for chronic scars and the required high doses of highly concentrated iodine-based contrast agents.

Real-time integration of imaging-derived scar at the beginning of the ablation procedure enables the operator to focus high-resolution mapping on scar sites harbouring potential VT substrates. Real-time integration of CCT-derived anatomic information on coronary vessels and phrenic nerve course may be helpful for epicardial VT ablation.

Cardiac computed tomography and CMR may be used to aid or guide ablation, but knowledge of the monomorphic sustained VT substrate in various structural heart diseases and the capability of imaging modalities to visualize this substrate are crucial. High registration accuracy of data sets (imaging, EAM data) is required and needs to be confirmed. Promising first results to localize crucial VT channels have been published for ICM and ACM using processed CMR and CCT data, complementary to mapping information.^79,80^ However, further studies are required.

### 5.1. Role of cardiac computed tomography and magnetic resonance imaging in ventricular tachycardia ablation

#### 5.1.1. General recommendations

**Figure 5:**
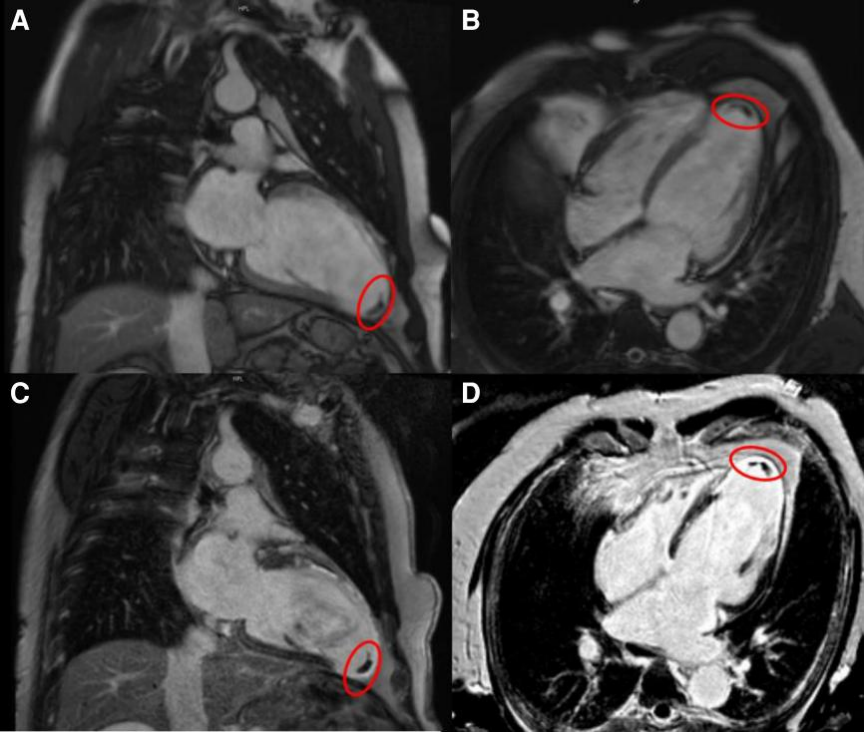
Cardiac magnetic resonance of a patient with history of anterior MI prior to VT ablation in cine (*A*, *B*) and LGE (*C*, *D*) (circle: thrombus in the LV apex). LGE, late gadolinium enhancement; MI, myocardial infarction; VT, ventricular tachycardia.

If LV endocardial ablation is planned, pre-procedural assessment for the presence of LV thrombi is warranted. Cine CMR and LGE have been shown to be superior to transthoracic echocardiography for the detection of LV thrombus (*Figure 5*). Late gadolinium enhancement provides a better diagnostic accuracy than cine CMR in the detection of laminated mural thrombi.^81,82^ Cardiac computed tomography has also been used, but studies comparing the diagnostic accuracy of CT for the exclusion of LV thrombus with other imaging modalities are scarce.^83^

#### 5.1.2. Epicardial mapping and ablation

Cardiac computed tomography and CMR may be useful for the planning of the optimal epicardial access. Cardiac computed tomography can detect anatomic variations of thoracic and abdominal structures that are at risk during subxiphoid puncture. Cardiac computed tomography is the gold standard for the assessment of epicardial fat distribution and thickness. A thick fat layer can attenuate voltages and can prevent effective RF lesions. Cardiac computed tomography can be also useful for accurate landmark settings, which facilitates real-time integration of cardiac and extracardiac structures.^84,85^ Cardiac computed tomography can accurately delineate the course of the coronary arteries in 74–85% of patients and the pericardiophrenic bundle. Accurate segmentation and integration in the setting of epicardial ablation may obviate the need for repeated coronary angiography and guide high-output pacing for phrenic nerve localization.

Cardiac magnetic resonance may delineate epicardial anatomic structures and may allow appropriate epicardial target identification. Thereby, the best epicardial access route may be indicated. In regard to spatial resolution, CCT has benefits over CMR. Epicardial adhesions have not been adequately identified with either technology.

### 5.2. Post-myocardial infarction cardiomyopathy ventricular tachycardia ablation

#### 5.2.1. Scar detection

In post-MI patients, there is a significant body of evidence demonstrating a correlation between CMR-defined scar and low-voltage areas identified by EAM.^2,86–92^ Bipolar and unipolar voltage amplitudes tend to decrease progressively with increasing scar transmurality and are affected by scar heterogeneity.^93^ The commonly used bipolar voltage cut-off of 1.5 mV has been shown to underestimate the size of non-transmural scars.^90^

**Figure 6:**
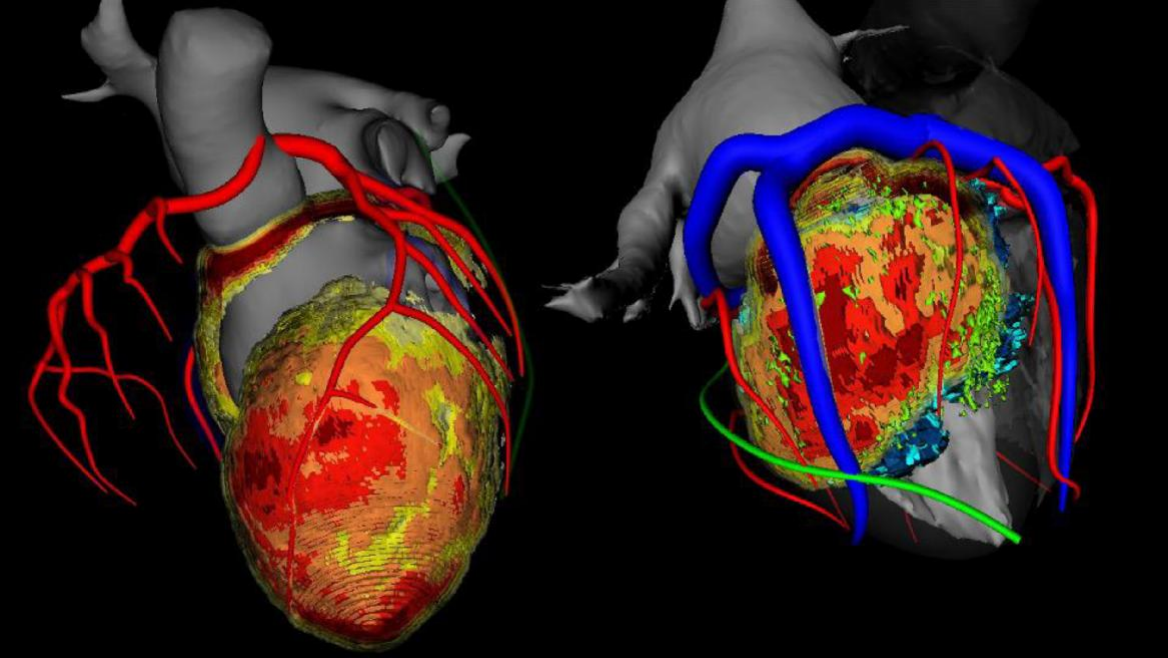
Three-dimensional reconstruction of cardiac chambers with colour-coded information on regional wall thickness using the InHeart technology. Critical anatomical structures (coronary arteries and veins) are visualized to guide the ablation procedure. Colour coding of LV depicting myocardial thickness: dark red: 1** **mm thickness, orange: 3** **mm thickness, and yellow: 4** **mm thickness; coronary arteries in red, coronary venous system in blue, and left phrenic nerve in green. LV, left atrial.

Contrast-enhanced multidetector CT (MDCT) is an alternative imaging technique for delineating post-infarct scars by assessment of myocardial wall thinning. Small observational studies have shown a moderate-to-good correlation between areas of wall thinning <5 mm on MDCT and bipolar voltages below 1.5 mV on EAM.^5,94–96^ In one study, severe wall thinning <2 mm was associated with the presence of transmural scar.^97^ Cardiac computed tomography allows also for the detection of myocardial calcification and fat^98,99^ (*Figure 6*).

#### 5.2.2. Real-time integration of imaging-derived scar

Real-time integration of LGE- and/or MDCT-defined scar during EAM has been successfully performed allowing to focus mapping and ablation.^1,2,5,79,92,94–97,100,101^ The use of image integration has been associated with a shorter procedural time^1,92,102^ and a shorter fluoroscopic time^1^ in non-randomized studies including post-MI or mixed cohorts of patients with scar-related VT. Improved procedural outcome has been reported if compared to historical cohorts without available imaging^1,2,92^, but data are inconsistent.^102^

#### 5.2.3. Cardiac imaging of ventricular tachycardia channels

It has been demonstrated that sites critical for VT maintenance were located in areas with transmural scar and core–border transition zones.^2,87–91^ In one study, heterogeneous conduction channels identified by LGE coincided spatially with conduction channels identified by EAM.^88,103–105^ However, these findings could not be confirmed by others.^106^ The feasibility of CMR-guided ablation based on LGE-derived pixel SI maps integrated into the EAM to target the potential VT substrate has been recently evaluated.^1^ Results showed shorter procedure duration and shorter fluoroscopy and RF delivery times in the CMR-guided group.

The ability of MDCT to identify potential post-infarct VT substrates is also under investigation with promising first results. Studies have shown that the majority of electrograms compatible with slow conduction as surrogate for the VT substrate were located in areas of wall thinning.^5,95–97,100^ The majority of ablation target sites were located in CCT-imaged VT channels, defined as corridors of abnormal but more preserved wall thickness than the surrounding edges^79,96^ (*Table 7*).

**Table 7:** Advice table for the use of CCT and CMR in ischaemic VT ablation procedures

Advice for the use of CCT and CMR in ischaemic VT ablation procedures	Strength of advice	Imaging modality	CT specifications	MR specifications
(1) Pre-procedural imaging is advised to rule out intracavitary ventricular thrombus	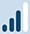	CMR or CCT	i.v. contrast injection and imaging in arterial and delayed phase	Early and late gadolinium enhancement sequences/LGE, steady-state free precession sequence (cine)
(2) Pre-procedural imaging may be appropriate to determine scar location	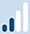	CMR or CCT	i.v. contrast injection and late iodine enhancement	LGE
(3) Pre-procedural imaging may be appropriate to determine scar transmurality	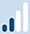	CMR or CCT	i.v. contrast injection and late iodine enhancement	LGE
(4) Pre-procedural imaging (CMR) may be appropriate to determine core–border zone transition	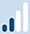	CMR	—	LGE
(5) Post-processing of imaging-derived scar (VT substrate) and integration into 3D mapping system may be appropriate to aid or guide VT ablation	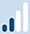	CMR or CCT	i.v. contrast injection arterial phase and late iodine enhancement	LGE

CCT, cardiac computed tomography; CT, computed tomography; CMR, cardiac magnetic resonance imaging; LGE, late gadolinium enhancement; MR, magnetic resonance; VT, ventricular tachycardia.

### 5.3. Non-ischaemic cardiomyopathy ventricular tachycardia ablation

#### 5.3.1. Pre-procedural imaging/planning

**Figure 7:**
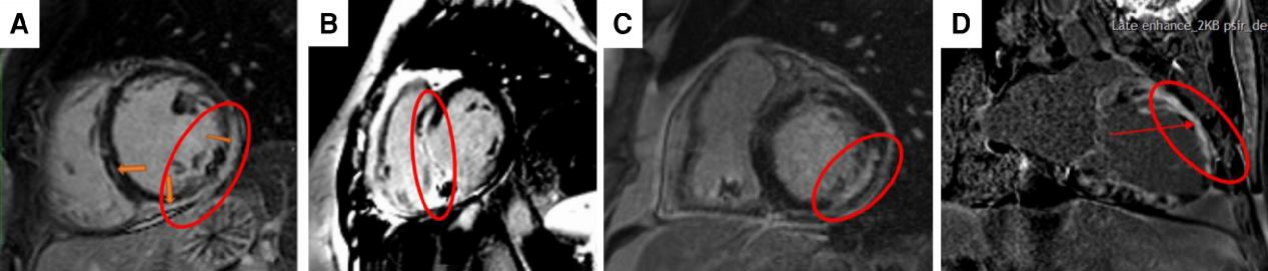
Different locations of LGE in non-ischaemic (*A–C*) and ischaemic (*D*) cardiomyopathy. Circles and arrows indicate the location of LGE:*A* subepicardially (in a patient after myocarditis), *B* intramural septal (in a patient with documented cardiac sarcoid), *C* intramural non-septal, *D* transmural/subendocardial in a patient with history of anterior transmural MI. Access route and ablation options could include epicardial access (*A*), bipolar septal ablation (*B*), bipolar endocardial–epicardial ablation (*C*), and primary endocardial access (*D*). LGE, late gadolinium enhancement; MI, myocardial infarction.

Cardiac magnetic resonance is a helpful tool in the diagnostic work-up of patients with unclear aetiology of VTs^107^ and is recommended in the European Society of Cardiology guidelines in all patients with non-ischaemic dilated cardiomyopathy.^108,109^ It is the gold standard for measuring LV and right ventricular (RV) volumes and ejection fraction. It also provides tissue characterization and may suggest the cause of ventricular dysfunction. Cardiac magnetic resonance is a valuable tool for the diagnosis of cardiac sarcoidosis, which is associated with a poorer outcome after ablation.^110^ If active myocardial inflammation is suspected and VTs can be temporarily controlled by antiarrhythmic drugs, catheter ablation should be postponed.^111^ 18F-Fluordeoxyglucose Positron Emission Tomography is the best clinically available tool for imaging myocardial inflammation.^112^ Cardiac magnetic resonance findings are limited by a relatively low specificity to distinguish scar from active inflammation.^112^ Areas with LGE on CMR have been associated with the VT substrates in different non-ischaemic cardiomyopathy (NICM) aetiologies, and the specific location can determine the access to the substrate^113,114^ (*Figure 7*). The role of LGE for individual risk stratification for sudden cardiac death has not been conclusively established. However, there is evolving evidence that amount and location of LGE on CMR are associated with ventricular arrhythmia in different non-ischaemic aetiologies^115–117^, and future studies will address this topic also including artificial intelligence and modelling algorithms.

Data suggest that VTs in patients with NICM are related to areas with LGE on CMR.^114,118^ Scar distribution and location can be highly variable.^119,120^ Accordingly, pre-procedural LGE-CMR is advised to identify scar location, scar transmurality, and scar depth to determine the best access and ablation strategy of the potential VT substrate.^87,121–124^ Specifically for deep intramural scar locations, the need for additional techniques and bail-out strategies, such as transcoronary or transvenous alcohol ablation or bipolar ablation, may be anticipated from pre-procedural imaging. Late acquisition CCT may allow depiction of the coronary venous anatomy and potentially select targets for ethanol ablation.

#### 5.3.2. Real-time integration of imaging-derived scar

Integration of the segmented scar from pre-procedural LGE into 3D mapping systems can be useful also in NICM patients with VT.^125^ Accurate delineation of non-ischaemic scars by EAM, in particular in the case of intramural location, has important limitations.^84,123,126^ Accordingly, if EAM is inconclusive for intramural scar detection, integration of the segmented scar can support ablation.^123^ For imaging-guided ablation, high integration accuracy needs to be achieved to prevent damage of viable myocardium.

Data on CCT for scar delineation in NICM are scarce. Contradictory and discouraging reports regarding the relationship between (rarely observed) wall thinning (<5 mm) and low-voltage areas^94,97^ have been published. In contrast, first experience with delayed enhancement on CCT (late iodine enhancement) is promising. In a small series of 19 patients with NICM, delayed enhanced CCT could predict low-voltage areas with a sensitivity of 78%, suggesting a potential role for both pre-procedural planning and intraprocedural scar localization.^95,127,128^

#### 5.3.3. Cardiac imaging for substrate-based ablation

Data on specific scar characteristics associated with VT in NICM are not available, and accurate delineation of non-ischaemic scars by imaging has important limitations.^126^ However, VT-related sites have been colocalized with LGE-derived scar features. In one small series, all concealed entrainment sites and 77% of VT termination sites were located in areas with ≥75% scar transmurality and in areas of core–border zone transition.^87^ In contrast, in a second series of a heterogeneous group of Dilated Cardiomyopathy patients (one-third with cardiac sarcoidosis), 15 of 18 VT-related sites occurred in areas of 25–75% scar transmurality.^91^ Inconsistency may be explained by the different patient cohorts and the different image processing methods for scar delineation. Further research on specific VT substrates and the optimal imaging modalities for the heterogeneous population of NICM is required and needs to be evaluated in larger patient cohorts (*Table 8*).

**Table 8:** Advice table for the use of CCT and CMR in non-ischaemic VT ablation procedures

Advice for the use of CCT and CMR in non-ischaemic VT ablation procedures	Strength of advice	Imaging modality	CT specifications	MR specifications
(1) Pre-procedural imaging is advised to rule out intracavitary ventricular thrombus	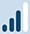	CMR or CCT	i.v. contrast injection and imaging in arterial and delayed phase	Early and late gadolinium enhancement sequences/LGE, steady-state free precession sequence (cine)
(2) Pre-procedural imaging is advised to determine scar location	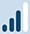	CMR or CCT	i.v. contrast injection and late iodine enhancement	LGE
(3) Pre-procedural imaging may be appropriate to determine scar transmurality	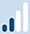	CMR or CCT	i.v. contrast injection and late iodine enhancement	LGE
(4) Pre-procedural imaging may be appropriate to determine intramural scar location	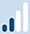	CMR	i.v. contrast injection and late iodine enhancement	LGE
(5) Pre-procedural imaging may be appropriate to identify areas of fibrofatty replacement in ARVC	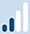	CCT	i.v. contrast injection and imaging in arterial phase	Cine, black-blood sequences, LGE
(6) Whether post-processing imaging-derived scar (VT substrate) and integration into 3D mapping system is useful to aid or guide VT ablation is uncertain	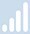	CMR	i.v. contrast injection arterial phase and late iodine enhancement	LGE

ACM, arrhythmogenic cardiomyopathy; CCT, cardiac computed tomography; CMR, cardiac magnetic resonance imaging; CT, computed tomography; LGE, late gadolinium enhancement; MR, magnetic resonance; VT, ventricular tachycardia.

### 5.4. Arrhythmogenic right ventricular cardiomyopathy ventricular tachycardia ablation

#### 5.4.1. Pre-procedural planning

The role of pre-procedural CMR for scar detection to optimize EAM has not been specifically addressed in arrhythmogenic right ventricular cardiomyopathy (ARVC), and data regarding the correlation between the VT substrate and scar localization are sparse.^129^ A significant correlation between abnormal epicardial right ventricular EGMs and standardized LGE-CMR SI *z*-scores has been reported.^130^ In another study, dense scar areas and VT-related sites as identified by EAM correlated better with CMR abnormalities when regional strain-analysis and LGE findings were combined.^131^ Whether these findings may help to predict the need for epicardial access requires further studies.

Fibrofatty replacement can also affect the LV. Left ventricular intramyocardial fat was present on MDCT imaging in the majority^132^ of patients fulfilling the modified Task Force Criteria for ARVC. The most affected regions were lateral, inferior, and apical LV segments with a lower fat burden compared to the RV. Although CCT-derived LV fat was associated with abnormal electrogram characteristics, voltage mapping could not accurately delineate LV fat areas. Accordingly, pre-procedural CCT may be useful to focus mapping on CCT-derived fat areas.^133^

#### 5.4.2. Real-time integration of imaging-derived fibrofatty areas

Segmented MDCT-derived intramyocardial fat can be integrated into EAMS and may guide mapping to the area of interest. In a series of 16 patients with ARVC, 80% of local abnormal ventricular activity electrograms were located within areas of intramyocardial fat on CCT.^134^ Homogeneous areas of intramyocardial fat may not necessarily be arrhythmogenic. In a cohort of 17 patients with ARVC, areas with CCT-derived high right ventricular tissue heterogeneity, which may better reflect the fibrofatty replacement, could detect areas with late potentials as surrogate for a VT substrate with high accuracy.^16^ Whether these CCT-derived and post-processed data will impact ablation outcome requires further studies.

### 5.5. Optimal computed tomography imaging modalities for patients with ventricular tachycardia

Cardiac computed tomography angiography can be used to detect significant coronary artery disease, chronic MI by using wall thickness as reference,^135^ to rule out LV thrombus,^136^ and to quantify epicardial fat^137^ and may help to visualize the large cardiac vessels and the pericardiophrenic bundle.^138^ A late acquisition can be obtained 7–10 min after contrast injection with the same prescription but with lower tube current and voltage (e.g. 80–100 kV) to increase the contrast-to-noise ratio and limit radiation dose in order to identify location and distribution of scar and to calculate ECV^139,140^ (*Table 7*).

### 5.6. Optimal cardiac magnetic resonance imaging modalities for patients with ventricular tachycardia

Cardiac magnetic resonance scan protocols focus on cardiac morphology, function, and tissue characterization in the work-up for the underlying aetiology. Cine images allow to evaluate wall motion and to quantify right and left volume and function.^141^ This data set allows to identify the presence of wall motion abnormalities suspicious for ICM vs. NICM^142^ and to distinguish between dilated, hypertrophic, or arrhythmogenic phenotypes.^142–144^ Cine images can be used for epicardial fat quantification. Mapping allows for pre-contrast tissue characterization and for the differential diagnosis of NICM subtypes. Black-blood T1w images, T1 mapping, black-blood T2w, and T2 mapping allow to identify fat infiltration, interstitial fibrosis, and oedema, respectively. Late gadolinium enhancement images should be obtained using 2D or 3D segmented inversion recovery gradient echo with the addition of post-contrast T1 mapping at least 10 min after the injection of gadolinium-based contrast agent.

### 5.7. Important considerations for the use of cardiac computed tomography and cardiac magnetic resonance in ischaemic ventricular tachycardia ablation procedures

(1) Cardiac computed tomography and CMR can detect LV thrombus and may be more accurate compared to transthoracic echo including echo contrast medium. Late gadolinium enhancement provides a better diagnostic accuracy than cine CMR in the detection of laminated mural thrombi.^81,82^ Data on the diagnostic accuracy of CCT compared to other imaging modalities are scarce.^83^
(2), (3), (4) Late gadolinium enhancement and CCT using late iodine enhancement can identify myocardial scar/fibrosis areas. Late gadolinium enhancement is the gold standard for identifying myocardial areas with different degrees of fibrosis. Late iodine enhancement CCT and myocardial thickness may identify areas with myocardial scar. Cardiac magnetic resonance and CCT may identify conducting channels within scar areas that may serve as diastolic conducting pathways. Cardiac magnetic resonance may also identify scar border zone depending on definition.
(5) Integration of CMR- or CCT-derived VT substrate information has been tested to aid or guide VT ablation. Cardiac magnetic resonance and CCT may be helpful in identification of VT channels in ICM. These channels may serve as targets for imaging-aided or imaging-guided VT ablation. Currently, randomized studies testing imaging-guided VT ablation are underway.

### 5.8. Important considerations for the use of cardiac computed tomography and cardiac magnetic resonance in non-ischaemic ventricular tachycardia ablation procedures

(1) Cardiac computed tomography and CMR can detect LV thrombus and may be more accurate compared to transthoracic echo including echo contrast. Late gadolinium enhancement provides a better diagnostic accuracy than cine CMR in the detection of laminated mural thrombi.^81,82^ Data on the diagnostic accuracy of CCT compared to other imaging modalities are scarce.^83^
(2), (3), (4) Late gadolinium enhancement and CCT using late iodine enhancement can identify myocardial scar/fibrosis areas in patients with non-ICM. Late gadolinium enhancement is the gold standard for identifying myocardial areas with different degrees of fibrosis including intramural location of scar areas in NICM. Late iodine enhancement CCT may identify areas with myocardial scar.
(5) Identification of areas with fibrofatty infiltration in patients with ACM is the domain of CCT. These areas may correspond to sites of abnormal electrograms related to VT circuits.
(6) Only limited data exist on integration of imaging-derived non-ischaemic VT substrate information on outcome of VT ablation. Future studies are needed.

## 6. Imaging for detection of ablation-related complications

Imaging can be exceptionally helpful in detection and risk characterization of ablation-induced complications. Computed tomography is the most commonly used modality as it is readily available and easier and faster to use in most ablation centres. In general, CT and MRI may be used to detect and classify oesophageal complications and PV stenosis after AF ablation as well as cerebral ischaemia and stroke after any left-sided ablation procedure. For characterization of vascular complications or detection of active vascular/access site bleeding, mostly CT imaging has been used. For documentation of oesophageal perforation, oral contrast application is helpful and therefore CT has been used more commonly in this scenario (*Table 9*).

**Table 9:** Imaging for detection/classification of complications

Imaging for detection/classification of complications		
	CCT	CMR
(1) Atrio-oesophageal fistula, oesophageal perforation	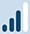	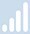
(2) Vascular complications, active bleeding	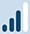	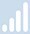
(3) Stroke, cerebral ischaemia	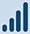	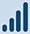
(4) PV stenosis	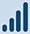	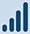

Imaging modalities and their role for detection of ablation-related complications.

CCT, cardiac computed tomography; CMR, cardiac magnetic resonance imaging; PV, pulmonary vein.

### 6.1. Oesophageal perforation after atrial fibrillation ablation

Both RF- and laser-induced heat and cryoballoon-based cooling may extend beyond the atrial myocardium and result in collateral damage to adjacent structures. Esophago-atrial fistula (or atrio-esophageal fistula, AEF) is a rare (≤ 0.2% of ablation procedures) but devastating complication with an estimated mortality of 60–80%.^145^ Symptoms include fever, chest pain, odynophagia, and neurological deficits. Due to the difficulty of diagnosis and the delay of presentation typically 2–6 weeks after the index ablation procedure, the occurrence of AEF is likely underestimated. Hence, rapid recognition and prompt treatment (usually by surgical repair) is of crucial importance.

**Figure 8:**
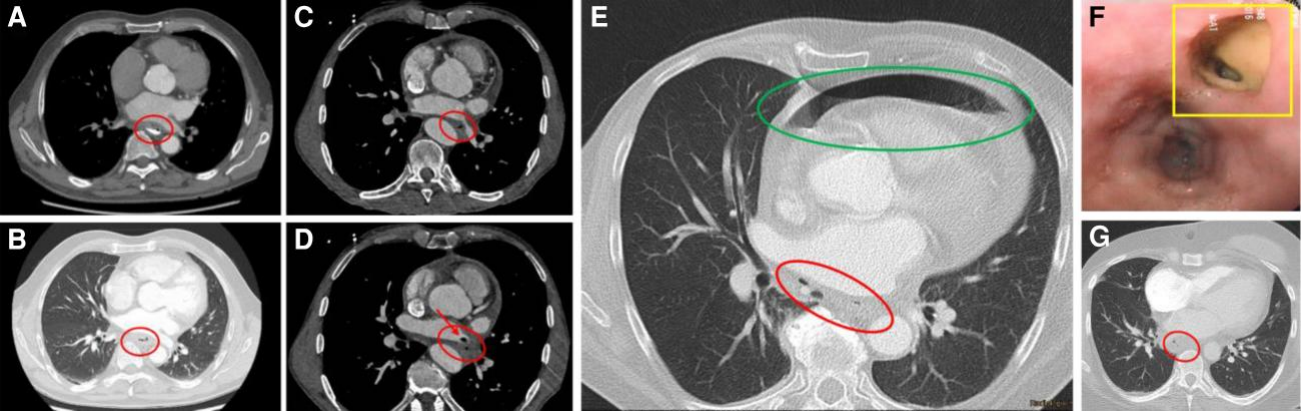
Characteristic CT (*A*–*E*, *G*) and endoscopy (*F*) findings in patients with *A* + *B*) oesophageal perforation, *C* + *D*) atrio-oesophageal fistula, *E*) oesophago-pericardial fistula, and *F* + *G*) perforating oesophageal ulcer. *A*) Air in mediastinum (red circle) with exit of water-soluble oral contrast into the mediastinum, *B*) i.v. contrast and documentation of air in mediastinum (red circle), *C* and *D*) air in mediastinum (red circle) and air and thrombus in the left atrium (arrow), *E*) i.v. contrast and identification of air in mediastinum (red circle) and pericardium (green circle), *F*) endoscopic finding 5 days after PV isolation with perforated oesophageal ulcer (yellow square), and *G*) corresponding CT with air in mediastinum (red circle). CT, computed tomography; PV, pulmonary vein.

Computed tomography of the chest is the preferred diagnostic test for AEF^146–149^ and to differentiate between AEF (72%) pericardial–oesophageal fistulas (14%) and oesophageal perforation (14%).^150,151^ In a published literature search,^145,149^ CT of the chest was the most common mode of diagnosis (68%) (*Figure 8*). Contrast CT of the chest was abnormal in 95/97 patients (98% of cases), although 7 cases (7%) required repeated testing. A repeat CT was diagnostic 4–12 days later, but it is unclear whether this may be due to inaccuracy of detecting oesophageal perforation on initial imaging (false negative) or progression of oesophageal injury during the course (true negative). Results of a recently published multicentre registry included 138 patients. Chest CT was used for diagnosis in 80.2%; overall mortality was 65.8% and highest if conservative management (89.5%) was pursued.^145^ An MRI study of the chest can be used alternatively, even though chest CT remains the diagnostic option of choice for fast and reliable evaluation including oral contrast medium application (*Figure 8*). It is critical that if AEF is suspected, no manipulation of the oesophagus (TOE or gastroscopy) should be performed prior to definitive diagnosis or exclusion of AEF. Air and material from the oesophagus may be introduced in the left atrium and can embolize via the atrium to the brain, potentially producing catastrophic neurological injury and death.^148^ Computed tomography scan of the chest is advised using i.v. and po water-soluble contrast and may need to be repeated (*Table 10*).

**Table 10:** Advice table for the use of CT and MR in the diagnosis and management of esophageal perforation

Advice for the use of CT and MR in the diagnosis and management of oesophageal perforation	Strength of advice	Imaging modality	CT specifications	MR specifications
(1) Early CT of the chest (including i.v. and po water-soluble contrast medium) is advised to diagnose or exclude oesophageal perforation in symptomatic patients within 6 weeks after AF ablation suspected to have oesophageal complications	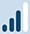	Chest CT	i.v. arterial phase contrast, po water-soluble contrast medium	
(2) Early imaging of the brain is advised in patients with atrio-oesophageal fistula and concomitant neurological symptoms to assess severity of brain damage and determine prognosis	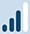	CMR or CCT		
(3) No manipulation of the oesophagus including oesophagogastroscopy or TOE is advised to prevent embolization of air and ingested material in patients with clinical suspicion before oesophageal perforation is excluded	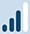			

AF, atrial fibrillation; CCT, cardiac computed tomography; CMR, cardiac magnetic resonance imaging; CT, computed tomography; MR, magnetic resonance; PV, pulmonary vein; TOE, transoesophageal echocardiography.

As neurologic symptoms frequently occur in patients with AEF, early brain imaging is mandatory to assess the severity of brain damage and to determine prognosis.^149^

Endoscopy is the gold standard for identifying and categorizing oesophageal thermal injury. Late gadolinium enhancement MRI of the oesophagus has been tested but appears to have a low positive predictive value when compared with endoscopy acutely after AF ablation. A negative LGE-MR appears to correlate well with no oesophageal thermal lesion detectable on post-ablation endoscopy.^152–154^

### 6.2. Important considerations for the use of computed tomography and magnetic resonance in the diagnosis and management of oesophageal perforation

1. Chest CT using intravenous and oral contrast medium as an emergency diagnostic procedure is key in patients presenting with classical symptoms of oesophageal perforation or AEF within the first 6 weeks after AF ablation. Computed tomography is readily available and can be performed as an emergency procedure. No data on the accuracy of MR in identifying oesophageal perforation are available.
2. Brain imaging (in most published cases CT) in order to determine severity and extent of brain damage is helpful in patients with neurological symptoms in patients presenting with AEF. Neurological symptoms are a negative prognostic indicator in patients with oesophageal perforation.
3. Any manipulation of the oesophagus in patients suspicious of having oesophageal perforation may aggravate oesophageal damage and must be avoided until oesophageal perforation has been excluded by CT [see also (1)]. Oesophagogastroscopy may be performed in patients with documented oesophageal perforation (usually using CO_2_ insufflation if at all needed) to diagnose site and extend of oesophageal damage and treatment.

### 6.3. Pulmonary vein stenosis after atrial fibrillation ablation

**Figure 9:**
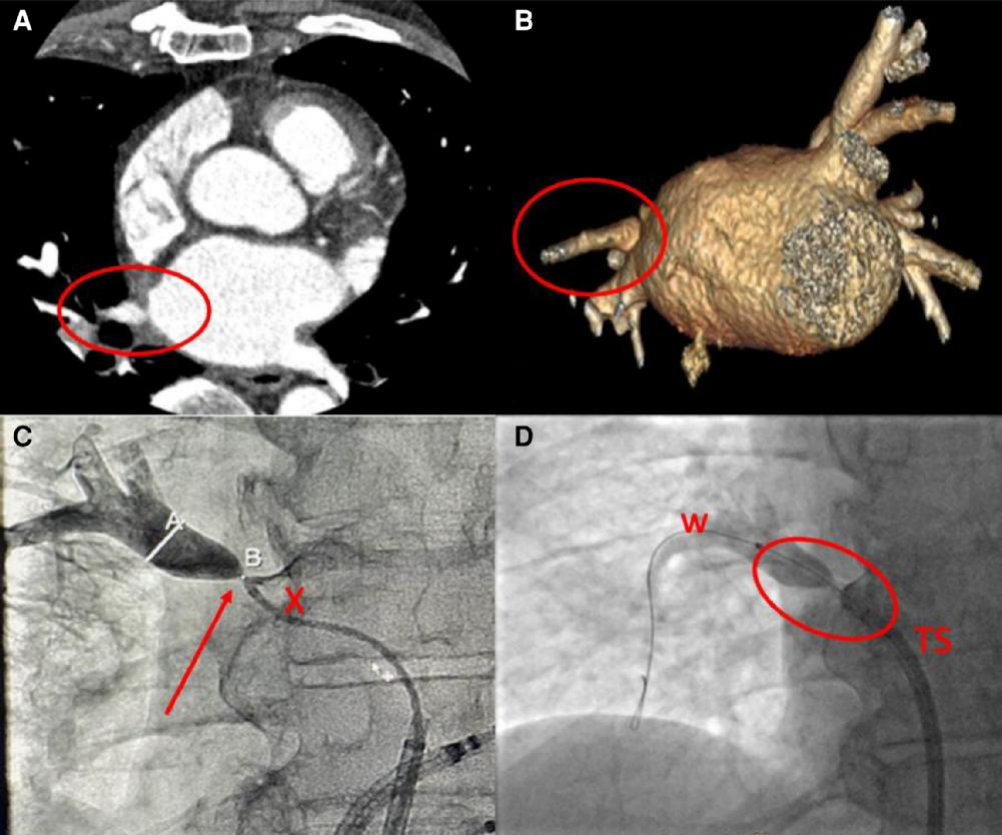
A patient with history of a prior PV isolation procedure and history of recurrent pneumonia in the right superior lobe following the procedure. *A*) CT showing high-degree stenosis of the right superior PV (circle), *B*) 3D reconstruction of the left atrium demonstrating a high-degree ostial stenosis of the right superior PV (circle), *C*) angiography of the right superior PV after transseptal access through a coronary diagnostic catheter (x) showing high-degree stenosis (arrow) (*A* indicates diameter of PV after stenosis, *B* site of stenosis), *D*) contrast injection into right superior PV after wiring (w) of stenosis (red circle) with transseptal sheath close to PV ostium. CT, computed tomography; PV, pulmonary vein.

**Figure 10:**
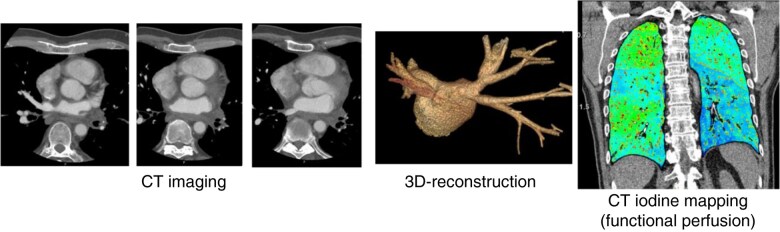
*A* and *B*) CT images of a patient with complete occlusion of the left inferior PV after prior PV isolation, *C* and *D*) post-stenting CT with stent open in left inferior PV, *E*) perfusion CT prior to stenting of the left inferior PV demonstrating a large perfusion defect in the left lung (blue colour). CT, computed tomography; PV, pulmonary vein

Pulmonary vein stenosis is a rare complication of PV isolation associated with significant morbidity.^155^ The incidence of this condition has been shown to decrease with the evolution and increasing experience with different ablation approaches.^156–159^ Many patients with PV stenosis remain asymptomatic and clinical symptoms in severe cases including dyspnoea, cough, fatigue, exertional chest pain, decreased exercise tolerance, and haemoptysis.^160^ Occurrence of these symptoms in patients following remote PV isolation procedure should alert physicians about this condition which might be misdiagnosed in about one-third of the patients.^155^ Non-invasive imaging modalities most commonly used in the diagnosis of PV stenosis include CT and MR angiography^158,161,162^ (*Figure 9*). Pulmonary vein stenosis severity is graded based on the degree of lumen diameter narrowing into mild (<50%), moderate (50–70%), and severe (>70%).^148^ Imaging to identify PV stenosis is usually performed post-ablation (several months following ablation) and/or upon occurrence of symptoms. Some studies report the use of routine screening for PV stenosis by repeated CT scans following ablation.^155,159^ Even with such an approach, the incidence of severe PV stenosis is low when contemporary ablation techniques are used. Therefore, this approach is no longer advised by the most recent consensus documents.^148^ According to published data, diagnostic accuracy of CT for the detection of PV stenosis is variable. Some of the studies report underestimation of severity of PV stenosis by CT.^156^ Lung perfusion cthe culprit PV in the case of multiple lesions^155,160^ (*Table 11*, *Figure 10*).

**Table 11:** Advice table for the use of CCT and CMR in the diagnosis and management of PV stenosis

Advice for the use of CCT and CMR in the diagnosis and management of PV stenosis	Strength of advice	Imaging modality	CT specifications	MR specifications
(1) CCT or CMR angiography is advised to diagnose PV stenosis in symptomatic patients following PV isolation	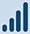	CMR or CCT	i.v. contrast injection and imaging in arterial and delayed phase	CEMRA
(2) Imaging is advised to plan and guide treatment of PV stenosis	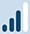	CMR or CCT	i.v. contrast injection and imaging in arterial and delayed phase	CEMRA
(3) Routine or serial imaging for asymptomatic PV stenosis is not advised	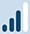		i.v. contrast injection and imaging in arterial and delayed phase	CEMRA

CCT, cardiac computed tomography; CEMRA, contrast enhancement magnetic resonance angiography; CMR, cardiac magnetic resonance imaging; CT, computed tomography; MR, magnetic resonance; PV, pulmonary vein.

**Figure 11:**
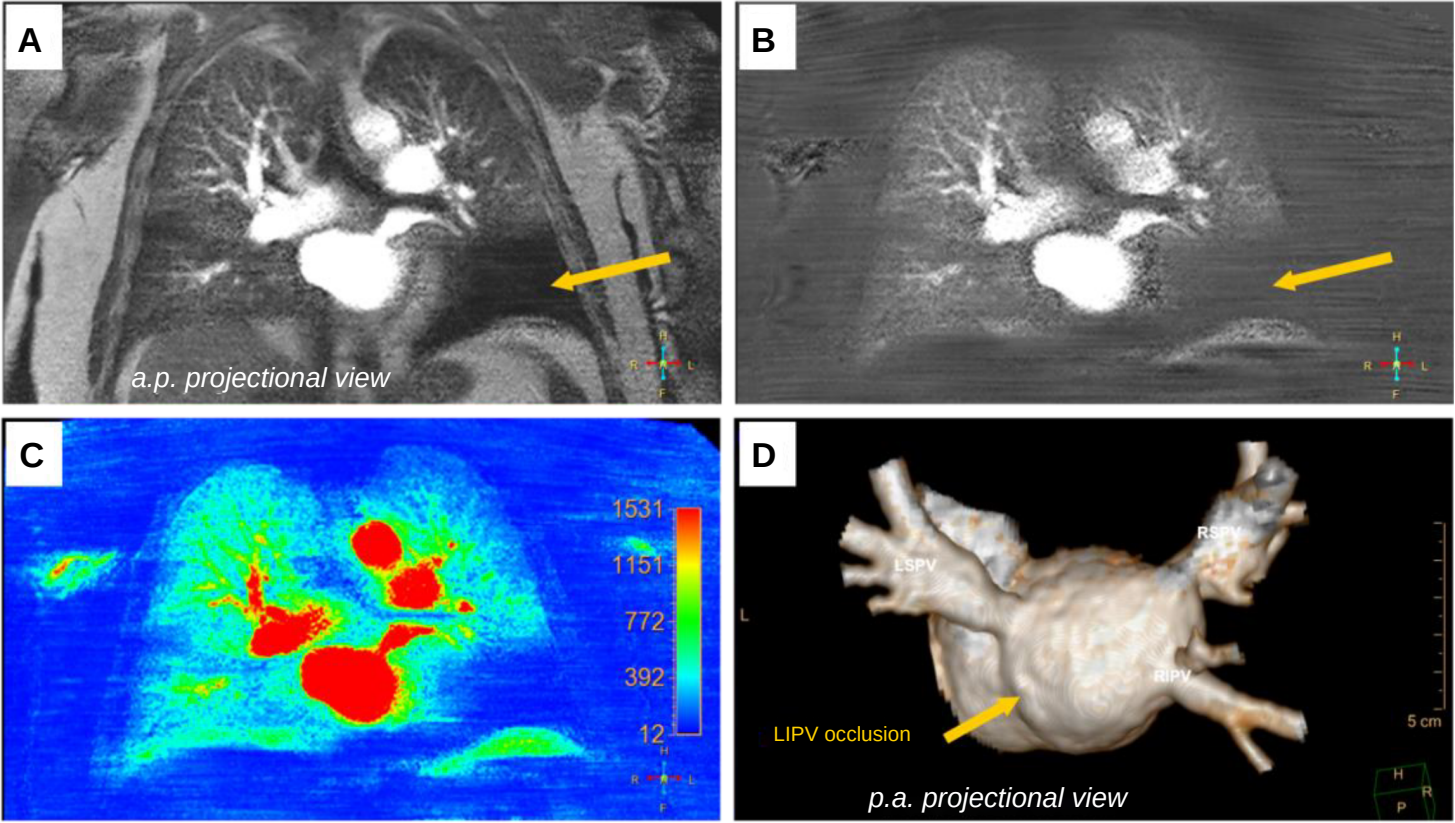
Pre-interventional CMR (cardiovascular magnetic resonance) pulmonary perfusion imaging and angiography for detection and characterization of pulmonary vein stenosis. *A, B, C*) CMR pulmonary perfusion imaging (anterior–posterior view) depicted a perfusion deficit of the left lower lung lobe (*A* still frame of original dynamic pulmonary perfusion; *B* still frame of dynamic pulmonary perfusion after background stationary tissue subtraction; *C* corresponding pseudo-coloured parametric map of quantitative CMR pulmonary perfusion analysis with SI maximum enhancement as the quantitative measure. *D*) Three-dimensional contrast-enhanced CMR angiography (posterior–anterior projectional view) revealed total ostial occlusion of the left lower pulmonary vein (arrow). CMR, cardiac magnetic resonance; SI, signal intensity.

Functional spectral perfusion mapping may be performed using CT (iodine mapping) or MR technologies and may help to classify effect of PV stenosis on lung perfusion similar to scintigraphic analysis. The potential benefit of CT may be the availability and the potential to identify structural effects of PV stenosis on lung tissue (*Figures 10* and *11*).

Non-invasive imaging with CT and MR can also be used to plan or guide treatment of PV stenosis. An early study used serial CT scanning to assess lesion progression over time.^161^ This approach does not seem to be justified as lesion progression beyond 3 months post-ablation was rare in the reported series. Role of MR angiography has not been studied in this regard. When balloon dilation or stenting is planned, the role of CT for pre-procedural stenosis assessment is questionable as it has been shown to be relatively insensitive to detect near-complete occlusions. In these cases, invasive angiography has been demonstrated to be superior in visualizing microchannels, the presence of which facilitates balloon dilatation or stenting.^163^

### 6.4. Important considerations for the use of computed tomography and magnetic resonance in the diagnosis and management of pulmonary vein stenosis

(1), (2) Non-invasive angiography using CT or MR is advised to diagnose or exclude PV stenosis in symptomatic patients after AF ablation. Usually, patients with stenotic PVs present with dyspnoea or multiple pulmonary infections/pneumonia. Imaging can help to quantify stenosis as well as locate completely occluded PVs. Comparison to pre-ablation imaging may be helpful. Imaging should include angiographic analysis of LA and PV anatomy. Functional spectral perfusion mapping using CT or MR can identify functional effects of PV stenosis and may be appropriate as alternative to ventilation–perfusion scintigraphy.^139,149,151,152^

(3) Routine post-procedural imaging to screen asymptomatic patients for PV stenosis or serial scanning to assess progression of identified PV stenosis is not indicated. Intervention of asymptomatic PV stenosis is generally not advised.

### 6.5. Neurological complications (stroke, Transient Ischemic Attack, and silent cerebral event/silent cerebral lesion) after ablation procedures

Cerebrovascular complication associated with left-sided ablation is an infrequent but potentially disabling event with a reported incidence of 0.5–5%.^164–167^ It typically occurs during or within the first 24 h of the procedure, with the majority of cases having been described in the first week after ablation.^168^ In the presence of new neurological symptoms compatible with cerebral ischaemia after the procedure, emergency brain imaging before initiation of any specific therapy is warranted. In patients who may be candidates for intravenous fibrinolysis, a non-contrast CT or MRI is sufficient to exclude the presence of intracerebral haemorrhage, allowing for immediate initiation of the treatment. Because the benefit of the therapy is time dependent, treatment should not be delayed for additional imaging. For patients who meet criteria for mechanical thrombectomy, non-invasive imaging of the intracranial arteries with CT angiography or MR angiography is advised during the initial imaging evaluation. Cerebral air embolism may be a potential reason for transient neurological symptoms and normal cerebral imaging. In patients presenting with neurological symptoms later after AF ablation (within 6 weeks) with or without other symptoms suggestive of oesophageal complication, it is advised to perform an early non-contrast CT or MRI of the brain and, in the case of pathological findings, a chest CT scan to exclude the presence of an AEF immediately.^149^

**Figure 12:**
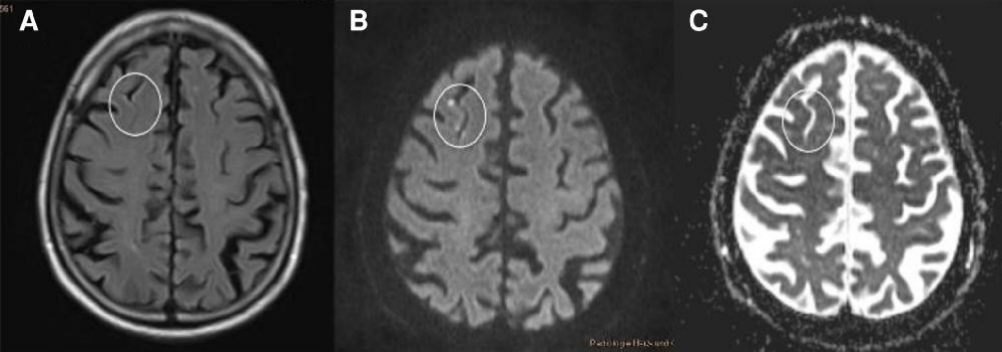
Silent cerebral events documented in a Patient 1 day after AF ablation on cerebral MR imaging: *A*) FLAIR sequence (no lesions), *B*) DWI with hyperintense lesions, and *C*) ADC map with corresponding hypointensity [differentiation between SCEs (FLAIR negative) and SCLs (FLAIR positive)]. ADC, apparent diffusion coefficient; DWI, diffusion-weighted imaging; FLAIR, fluid-attenuated inversion recovery; MR, magnetic resonance; SCL, silent cerebral lesion; SCE, silent cerebral events.

After ablation, a silent cerebral event (SCE) has been defined as the presence of an acute, new, asymptomatic cerebral ischaemic lesion on brain MRI. Silent cerebral event has been identified in 1.7–71% of patients after AF ablation and in up to 58% after ablation of left-sided ventricular arrhythmias.^169–172^ Cerebral ischaemia can be detected on MRI within minutes after its onset as a hyperintense diffusion-weighted imaging (DWI) lesion with a reduced apparent diffusion coefficient (ADC) map. On the contrary, fluid-attenuated inversion recovery (FLAIR) sequences become positive only later and depending on the lesion size. While some diffusion changes may be reversible, FLAIR-positive asymptomatic lesions seem to correspond to areas of brain scar and should therefore be called silent cerebral lesions (SCLs)^173,174^ (*Figure 12*). Because the clinical relevance of SCE/SCL remains unclear, to date no indication for routine brain MRI after left-sided ablation can be established^175,176^ (*Table 12*).

**Table 12:** Advice table for the use of CT and MR in the diagnosis and management of neurological complications

Advice for the use of CT and MR in the diagnosis and management of neurological complications	Strength of advice	Imaging modality	CT specifications	MR specifications
(1) Emergent brain imaging is advised in all patients with new neurological symptoms compatible with cerebral ischaemia after left cardiac chamber ablation before initiation of any specific therapy	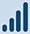	CT or MRI		
(2) In patients who are potential candidates for treatment with intravenous fibrinolysis, a non-contrast CT or MRI is advised to exclude intracranial haemorrhage	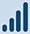	CT	Cerebral non-contrast CT	
(3) In patients who are potential candidates for treatment with mechanical thrombectomy, non-invasive imaging of the intracranial arteries is advised during the initial imaging evaluation	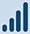	CT or MR	Angiography	Angiography
(4) In patients presenting with new neurological symptoms within 6 weeks of the ablation, emergent brain imaging is advised, followed in the case of pathological findings by a chest CT to evaluate for atrio-oesophageal fistula	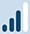	CT or MR		

CT, computed tomography; MR, magnetic resonance.

### 6.6. Important considerations for the use of computed tomography and magnetic resonance in the diagnosis and management of neurological complications

1. Brain imaging to diagnose and exclude intracranial haemorrhage and ischaemia is key for managing patients with neurological symptoms related to left cardiac chamber ablation. Magnetic resonance may be superior when looking for ischaemic defects but time from onset to imaging may influence modality and sensitivity.^160^
2. Intracranial haemorrhage must be ruled out before initiating intravenous fibrinolysis for acute cerebral ischaemia. Non-contrast CT or MR is comparably sensitive and specific.^160^
3. Non-invasive angiography of the intracranial arteries using CT or MR is advised in preparation of mechanical thrombectomy to diagnose anatomy and territory of arterial occlusion.
4. Neurological symptoms may be a first sign of AEF with embolism, and therefore, emergent brain imaging is advised in addition to chest CT to determine severity and extent of brain damage.

### 6.7. Complications related to vascular access

**Figure 13:**
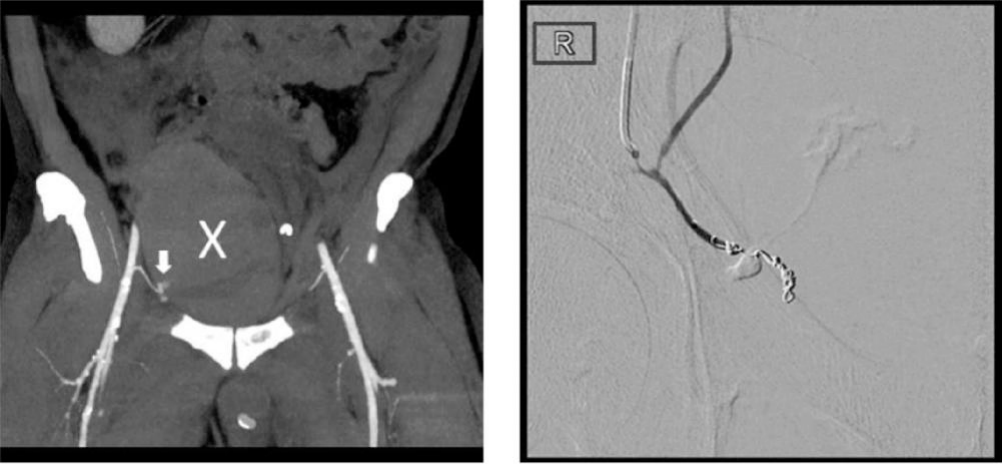
Left: CT scan with contrast showing active bleeding (arrow) and a large retroperitoneal haematoma (X) following vascular access for pulmonary vein isolation in an obese patient (BMI 32 kg/m²). Bleeding source was the right obturator artery which has a variant originated from the external iliac artery. Right: digital subtraction angiography post-embolization (coil). Courtesy of Dr. N. Thieme, Charité Universitätsmedizin, Berlin. BMI, body mass index; CT, computed tomography.

The most common complications in arrhythmia ablation are vascular access-related complications (VASC). Incidence is highest in AF and VT ablation and higher with transaortic vs. transseptal access.^177,178^ Vascular access-related complications include major haemorrhage, formation of a pseudo-aneurysm, arterio-venous fistula, and, rarely, aortic dissection. Patients with obesity, pre-existing vascular disease, female gender (smaller vessels), and advanced age are at higher risk.^179^ In general, ultrasound-guided puncture may be appropriate to reduce VASC in EP procedures.^180,181^ Haemodynamic instability, swelling, pulsatile mass, bruits, neurological symptoms, unusual or protracted pain at the puncture site, pelvis or leg (femoral access), or a relevant haemoglobin drop (≥2 mg/dL) following a procedure should trigger urgent imaging of the region. While ultrasound is easily accessible at bedside and the method of choice for diagnosis of pseudo-aneurysms, in patients with suspected haemorrhage, CT is advised for a precise diagnosis.^179,182^ Haematoma is identified by hypoattenuation, by swelling of adjacent muscle, and, subacutely, by a heterogeneous pattern (‘haematocrit sign’^179^) A bolus-triggered CT angiogram following application of iodinated contrast media detects the source of active bleeding (*Table 13*, *Figure 13*).

**Table 13:** Advice table for the use of CT and MR in the diagnosis and management of vascular and epicardial access complications

Advice for the use of CT and MR in the diagnosis and management of vascular and epicardial access complications	Strength of advice	Imaging modality	CT specifications	MR specifications
(1) CT imaging is advised to detect the active source and extent of haematoma in patients with suspected haemorrhage following vascular access	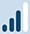	CT	CT with bolus-triggered iodinated contrast	_
(2) CT imaging is advised in the case of haemodynamic instability (in the absence of relevant pericardial effusion) or other clinical evidence of haemorrhage following subxiphoid epicardial access	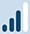	CT	Contrast CT of chest and abdomen	_

CT, computed tomography; MR, magnetic resonance.

### 6.8. Complications related to epicardial access

Epicardial access for ablation of ventricular and supraventricular arrhythmias is routinely performed by subxiphoid percutaneous puncture.^183^ Epicardial is often combined with endocardial catheter ablation to improve efficacy but is associated with an increased rate of acute complications (up to 13.7% for VT ablation).^183,184^ These include injury to the myocardium or epicardial vessels, RV puncture, intra-abdominal bleeding (hepatic puncture), phenic nerve injury, and RV pericardial fistulas.^183^ Pericardial bleeding and tamponade, the most common complications, should be established by echocardiography in the EP lab, and epicardial vessel injury by immediate coronary angiogram. Contrast CT allows for identification of the active bleeding site in unclear cases. In the case of haemodynamic instability in the absence of pericardial effusion or haemoglobin drop, intra-abdominal bleeding should be suspected and trigger immediate CT imaging.^185^ Due to lack of evidence, no advice can be given for the use of MRI in vascular and pericardial access-related complications.

### 6.9. Important considerations for the use of computed tomography and magnetic resonance in the diagnosis and management of vascular and epicardial access complications

1. In patients with suspected haemorrhagic complication related to vascular access, contrast CT can detect the active source and extent of haematoma. Routine imaging to rule out vascular complications is not advised.
2. In the case of haemodynamic instability and in the absence of relevant pericardial effusion after pericardial access, a contrast CT scan of the chest and abdominal region is advised to identify or rule out intra-abdominal or chest bleeding. In rare cases, damage to the epicardial coronary arteries or thoracic vasculature may complicate anterior pericardial puncture. The posterior access route has a higher incidence of complications as the abdominal space is punctured.^186^ Liver haemorrhage or damage may result from inadvertent puncture, especially in the case of right heart failure.

## 7. Future aspects, studies, and concepts

**Figure 14:**
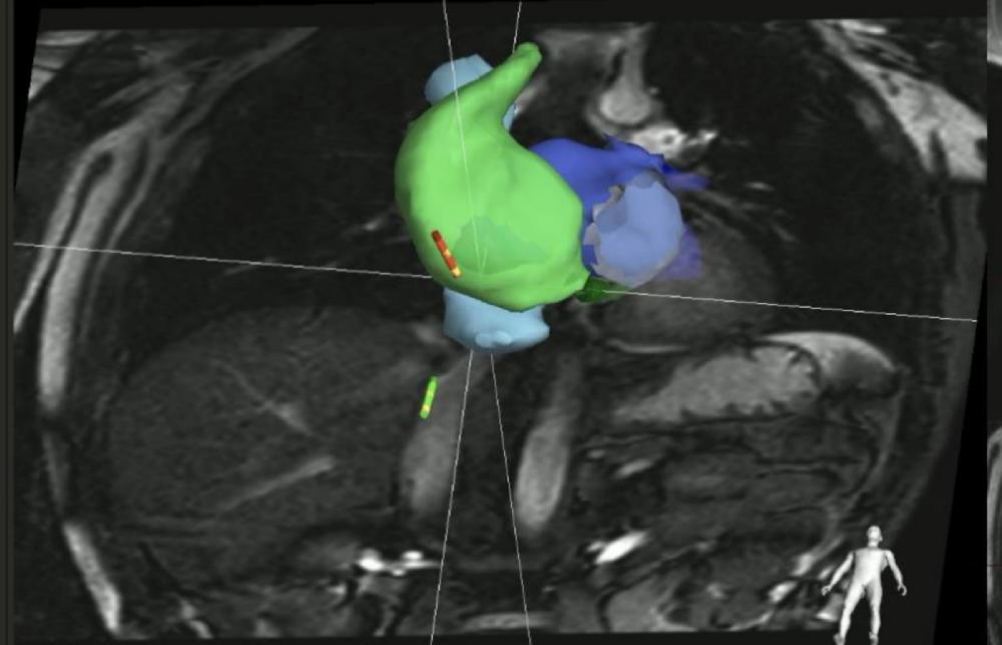
Interventional CMR-guided ablation procedure. Two catheters (green and red tip) are actively tracked and displayed on MR images and 3D reconstructed cardiac surfaces (green: right atrium, dark blue: left atrium, and light blue: superior and inferior vena cava). Courtesy of Leipzig Heart Center. CMR, cardiac magnetic resonance; MR, magnetic resonance.

In order to avoid the inherent inaccuracy, when imaging information is exported into another modality (3D mapping system) and the CCT/CMR maps have to be integrated (‘fused’) with landmarks in the 3D mapping, the next step has been to acquire image information in MRI and to perform the ablation within the MR scanner (*Figure 14*). This can be performed by interacting between a conventional EP lab and an MRI scanner (MRI suite) or entirely in an ‘interventional CMR lab’. Multiple hardware and software components of EP systems including diagnostic and ablation catheters were manufactured to fit the MR environment and have been tested in entirely CMR-guided procedures specifically for ablation to treat typical atrial flutter,^187^ but clinical benefit has to be proven. In an experimental set-up also, imaging and ablation for VTs have been reported showing the impressive possibilities that MRI is offering to the electrophysiologist like online lesion monitoring or thermometry as a tool to titrate energy delivery in different areas of the heart.^31^

Registration of multiple imaging modalities into the same mapping system may add benefit in some entities. In addition, novel imaging strategies and higher-resolution images may allow imaging of so far invisible structures that may gain importance as treatment targets (like myocardial fibre orientation or cardiac autonomic innervation). Also, the concept of imaging being used to follow-up and evaluate effect of atrial and ventricular ablations is interesting for future studies. We are facing a fascinating world of new insights and options including implementation of artificial intelligence as tool for future innovations.

## Data Availability

There was no data used to generate the manuscript as it is a consensus statement. All authors though have had complete access to the manuscript and all statements/advise generated in the manuscript was consented through all authors.
