# Correction to: Pre- and post-procedural cardiac imaging (computed tomography and magnetic resonance imaging) in electrophysiology: a clinical consensus statement of the European Heart Rhythm Association and European Association of Cardiovascular Imaging of the European Society of Cardiology

**DOI:** 10.1093/europace/euae166

**Published:** 2024-06-14

This is a correction to: Thomas Deneke, Valentina Kutyifa, Gerhard Hindricks, Philipp Sommer, Katja Zeppenfeld, Corrado Carbucicchio, Helmut Pürerfellner, Frank R Heinzel, Vassil B Traykov, Marta De Riva, Gianluca Pontone, Lukas Lehmkuhl, Kristina Haugaa, Andrea Sarkozy, Alessia Gimelli, Claudio Tondo, Sabine Ernst, Matthias Antz, Mark Westwood, Pre- and post-procedural cardiac imaging (computed tomography and magnetic resonance imaging) in electrophysiology: a clinical consensus statement of the European Heart Rhythm Association and European Association of Cardiovascular Imaging of the European Society of Cardiology, *EP Europace,* Volume 26, Issue 5, May 2024, euae108, https://doi.org/10.1093/europace/euae108

In the originally published version of this article, there was a typographical error in author Corrado Carbucicchio’s name.

This has been corrected.

